# An integrated GIS and remote-sensing framework for district-level dengue risk assessment in northern India: development of a spatial Dengue Risk Index and assessment of its spatial concordance with reported incidence

**DOI:** 10.1186/s41182-026-01096-1

**Published:** 2026-10-22

**Authors:** U. Venkatesh, Amjed Ali, Arshad Ahmed

**Affiliations:** Department of Community & Family Medicine, All India Institute of Medical Sciences (AIIMS), Gorakhpur, Uttar Pradesh 273008 India

**Keywords:** Dengue, Spatial epidemiology, Geographic Information System (GIS), Remote sensing (RS), Risk assessment

## Abstract

**Background:**

Dengue is an increasingly important vector-borne disease driven by climate variability, urbanization, environmental change, and population growth. Despite its rising burden in northern India, district-level spatial epidemiological frameworks integrating environmental suitability, transmission hazard, and population vulnerability remain limited. This study developed an integrated geographic information system (GIS)- and remote-sensing (RS)-based Dengue Risk Index (DRI) describing long-term spatial susceptibility to dengue in Gorakhpur district, Uttar Pradesh, and assessed its spatial concordance with reported dengue incidence.

**Methods:**

A GIS-based multi-criteria decision analysis (MCDA) framework incorporating the analytic hierarchy process (AHP) integrated 14 environmental, topographic, hydrological, and socio-demographic variables into three thematic components: the Environmental Suitability Index (ESI; six variables), Hazard Index (HI; five variables), and Vulnerability Index (VI; three variables). Relative humidity and the Normalized Difference Moisture Index were excluded after redundancy screening. Rainfall was derived from India Meteorological Department (IMD) 0.25° daily gridded data for 2025, elevation from the Copernicus 30 m DEM, and population density from WorldPop 2025 constrained estimates. Standardized (1–5) raster layers were integrated using the Weighted Linear Combination (WLC) approach to construct the DRI. Spatial concordance between block-level mean DRI and laboratory-confirmed dengue incidence in 2025 (225 cases, 20 blocks) was assessed using Pearson’s and Spearman’s correlation, linear and negative binomial regression, influence diagnostics, and global Moran’s I with Lagrange multiplier tests for spatial dependence.

**Findings:**

The DRI (1–5 relative scale) showed marked spatial heterogeneity, with higher values in the central and western parts of the district. Chargawan, which contains Gorakhpur city, was the only block classified as Very High risk, followed by Sahjanwa, Piprauli, and Jungle Kaudia (High), whereas Brahmpur was classified as Very Low risk. Overall, 32.87% of the district (1,090.2 km^2^) was classified as High or Very High risk. The single very high-risk block accounted for 48.9% of reported cases. However, concordance between block-level DRI and incidence was weak and not statistically significant (Pearson's *r* = 0.16, *p* = 0.49; Spearman's ρ = −0.003, *p* = 0.99; *R*^2^ = 0.03; incidence rate ratio per 0.1 DRI = 1.05, 95% CI 0.91–1.21). Neither incidence nor the regression residuals showed significant spatial autocorrelation (Moran's *I* = −0.13 and −0.17; *p* ≥ 0.44), so spatial regression was not required.

**Interpretation:**

The DRI provides a transparent, reproducible description of long-term spatial susceptibility to dengue that integrates environmental suitability, transmission hazard, and population vulnerability. Because its concordance with single-year, block-level incidence was weak and non-significant, it should not be interpreted as a predictor of dengue incidence or as a validated risk model. The framework may help district programs structure surveillance planning, but multi-year incidence, finer spatial units, and entomological data are needed before it is used to allocate interventions.

**Supplementary Information:**

The online version contains supplementary material available at https://doi.org/10.1186/s41182-026-01096-1.

## Introduction

Dengue has emerged as one of the fastest-growing mosquito-borne viral diseases and is now recognized as a major global public health challenge. Transmitted primarily by Aedes aegypti and Aedes albopictus, dengue has expanded rapidly because of climate change, accelerated urbanization, environmental degradation, globalization, increasing international travel, and human mobility, which have facilitated the spread of competent vectors beyond their traditional tropical distribution [1, 2]. Recent Global Burden of Disease (GBD) estimates reported approximately 58.96 million dengue cases globally in 2021, while the World Health Organization [3] documented more than 13.8 million reported cases and over 10,000 deaths in 2024, representing the highest annual burden recorded to date. These findings underscore the urgent need for evidence-based surveillance and geographically targeted prevention strategies [4, 5]. Temperature, rainfall, humidity, vegetation, land-use change, urban expansion, water-storage practices, and population density collectively influence mosquito habitat suitability and human exposure, while globalization and population mobility facilitate virus circulation across regions [6–8]. Consequently, dengue transmission exhibits pronounced spatial heterogeneity, underscoring the importance of spatial epidemiological approaches for identifying localized determinants of disease risk and supporting targeted public health interventions.

South Asia has become one of the world's fastest-growing dengue hotspots, where favorable climatic conditions, high population density, rapid urbanization, inadequate sanitation, and environmental degradation collectively sustain mosquito breeding and virus transmission [3, 9, 10]. India contributes substantially to the regional dengue burden, with transmission increasingly extending beyond metropolitan centers into peri-urban and rural communities [3, 11]. Within India, Uttar Pradesh is particularly vulnerable because of its humid subtropical climate, extensive river systems, seasonal monsoon rainfall, rapid urban growth, agricultural landscapes, and heterogeneous environmental conditions that favor Aedes mosquito proliferation [12–14]. Despite strengthened surveillance and vector-control programs, dengue outbreaks continue to exhibit considerable spatial variation, indicating that transmission risk is strongly influenced by local environmental and socio-demographic conditions [9, 15]. Gorakhpur district in eastern Uttar Pradesh represents an important yet comparatively understudied setting for dengue epidemiology [16, 17]. High annual rainfall, prolonged monsoon seasons, recurrent flooding, fertile alluvial plains, extensive agricultural landscapes, rapidly expanding urban settlements, and dense rural populations collectively create favorable ecological conditions for mosquito breeding [9, 12, 18]. Although the district has been extensively investigated for Japanese encephalitis and acute encephalitis syndrome, comparatively limited attention has been given to the spatial epidemiology of dengue despite increasing disease occurrence [19, 20].

Recent advances in geographic information systems (GIS), remote sensing (RS), and spatial epidemiology have substantially improved the understanding of vector-borne disease dynamics by integrating climatic, environmental, topographic, and socio-demographic information within geospatial analytical frameworks [21, 22]. Nevertheless, previous studies have primarily focused on individual analytical components, including environmental suitability, hotspot detection, climatic associations, or dengue prediction, rather than integrated spatial epidemiological frameworks [23, 24]. Furthermore, relatively few district-level studies have simultaneously integrated environmental suitability, transmission hazard, and population vulnerability within a unified GIS-based framework for dengue risk assessment, particularly in northern India [25–27]. Accordingly, this study developed an integrated geographic information system (GIS) and remote-sensing (RS)-based spatial epidemiological framework for district-level dengue risk assessment in Gorakhpur district, Uttar Pradesh. The framework integrates the Environmental Suitability Index (ESI), Hazard Index (HI), and Vulnerability Index (VI) to construct the Dengue Risk Index (DRI). The spatial concordance between the DRI and reported block-level dengue incidence was then assessed, and global spatial autocorrelation of the DRI, incidence, and model residuals was tested to determine whether spatial dependence needed to be accounted for in the DRI–incidence relationship. The DRI is intended as a description of long-term spatial susceptibility (dengue transmission suitability combined with hazard and vulnerability), rather than as a predictive model of incidence.

## Methodology

### Study design and analytical framework

**Fig. 1:**
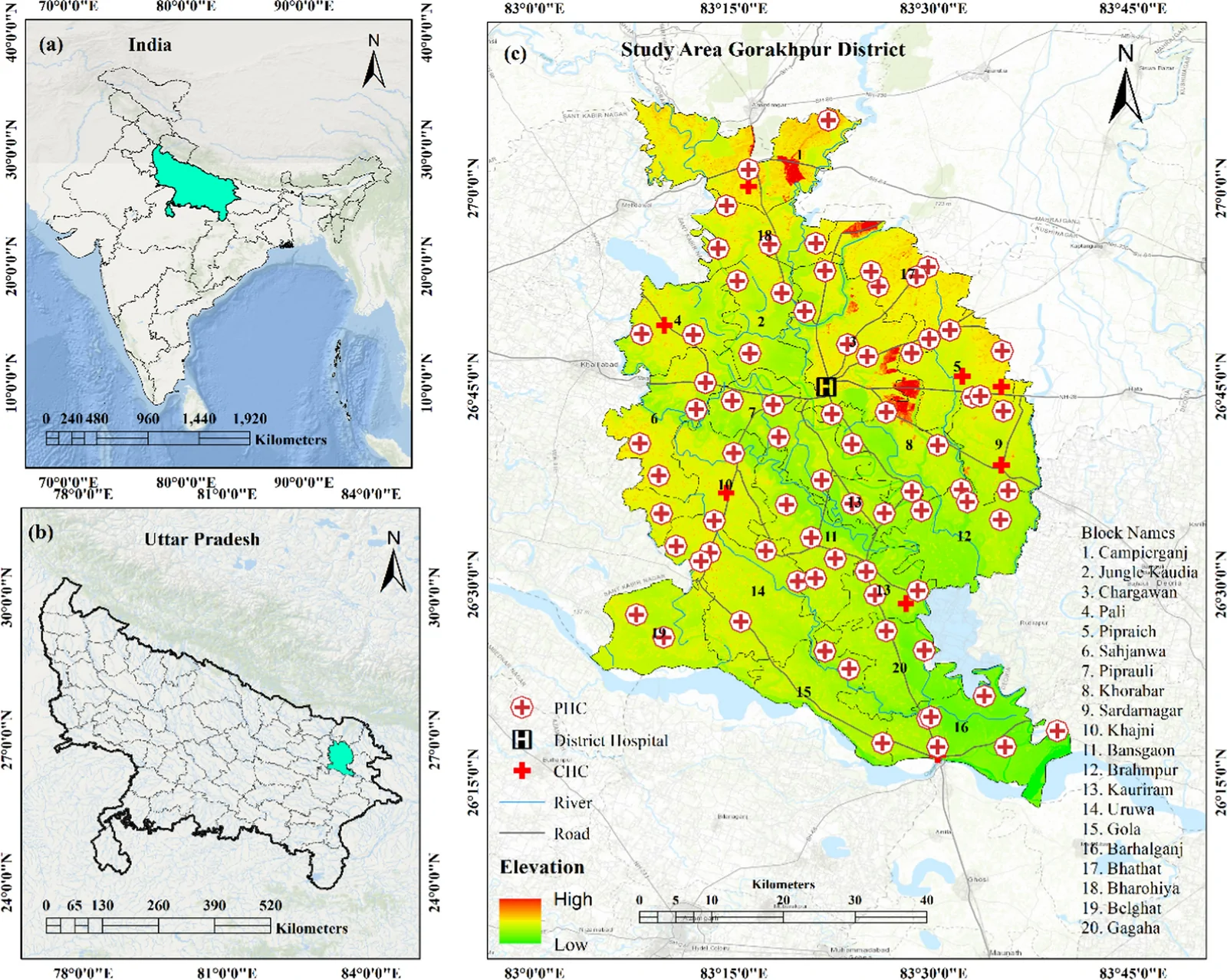
Study area and geographic location of Gorakhpur district, Uttar Pradesh, India. **a** Location of Uttar Pradesh within India; **b** location of Gorakhpur district within Uttar Pradesh; and **c** study area showing administrative block boundaries, PHCs, CHCs, the district hospital, road and river networks, and elevation

This retrospective ecological GIS-based spatial epidemiological study employed a GIS-based spatial risk assessment framework to assess dengue transmission risk in Gorakhpur district, Uttar Pradesh, India (Fig. 1). The framework integrated RS, GIS, MCDA, AHP, and the WLC approach to construct the DRI, which integrates environmental suitability, transmission hazard, and population vulnerability into a composite, relative measure of spatial susceptibility to dengue. Terminology is used consistently throughout: “dengue transmission suitability” refers to the environmental conditions summarized by the ESI; “spatial susceptibility” refers to the composite condition summarized by the DRI (suitability, hazard, and vulnerability combined); and “risk class” refers to the five relative DRI categories. “Dengue Risk Index” and “dengue risk assessment” are retained only as the names of the index and framework.

**Fig. 2:**
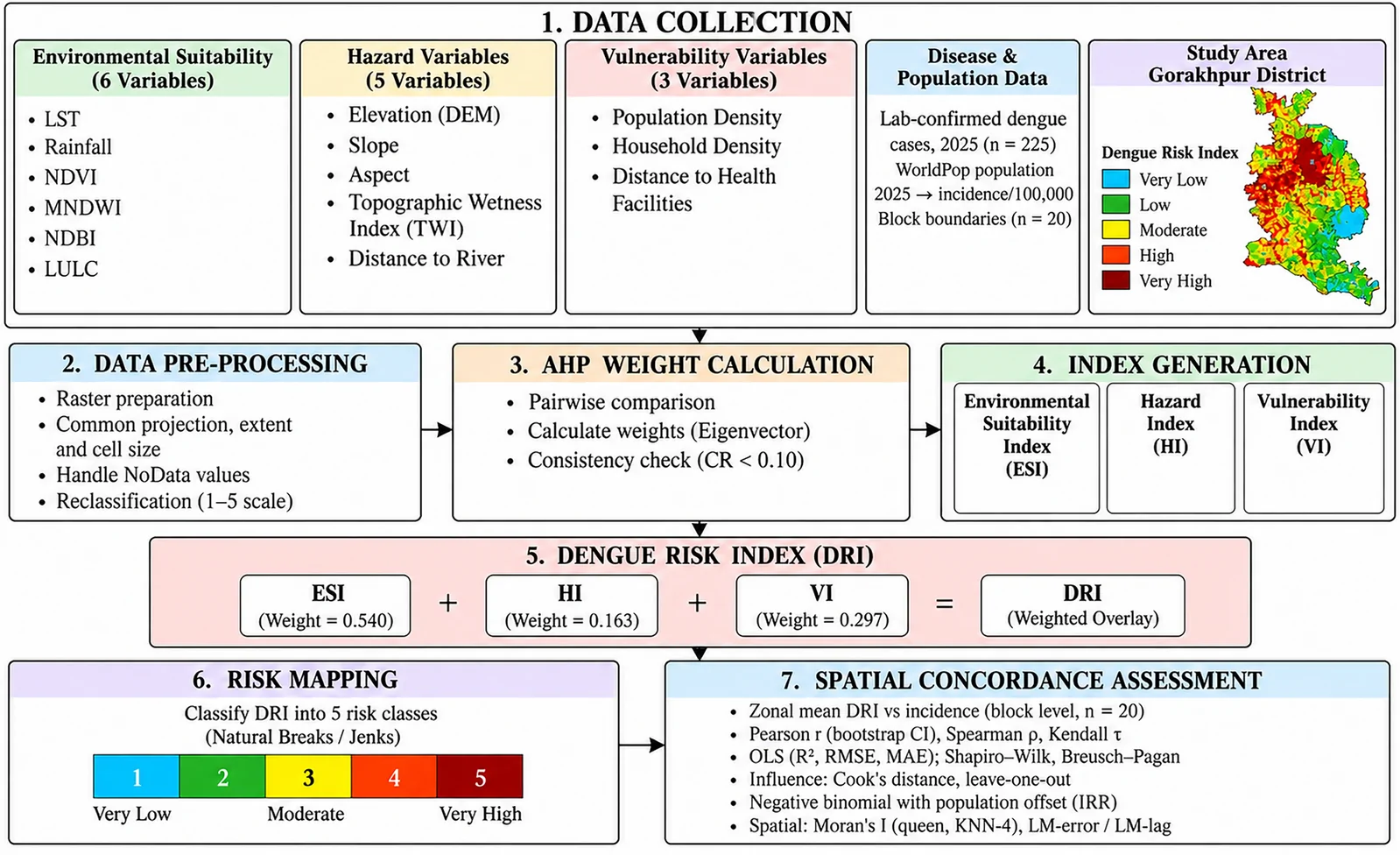
Methodological workflow for GIS-based spatial assessment of dengue risk in Gorakhpur District, Uttar Pradesh, India

Administrative blocks (Community Development Blocks) were selected as the spatial unit, because laboratory-confirmed dengue cases were routinely reported at the block level in the District Health Surveillance System. Block boundaries also correspond to the operational unit used for district-level surveillance and vector-control planning. Finer units such as PHC/CHC catchments or villages were, therefore, not used, because corresponding case data were unavailable at these spatial levels. The study covered the period from January to December 2025. Laboratory-confirmed dengue cases reported during 2025 were used for the spatial concordance assessment. Environmental variables were derived from cloud-free Landsat-8/9 imagery, rainfall was obtained from the India Meteorological Department (IMD) high-resolution daily gridded rainfall dataset [28], and static spatial datasets, including the DEM, river network, road network, healthcare facilities, and administrative boundaries, were considered temporally invariant throughout the study period. A total of 14 variables were classified into three thematic domains: environmental suitability (6 variables), hazard (5 variables), and vulnerability (3 variables) [29–31]. All variables were standardized, weighted using AHP, and integrated using the WLC approach to construct the ESI, HI, VI, and the final DRI. The overall analytical workflow is presented in Fig. 2.

### Spatial and epidemiological data collection

Spatial and epidemiological datasets representing the environmental, physical, and socio-demographic determinants of dengue transmission were compiled from multiple national and international sources to develop the Geographic Information System (GIS)-based dengue risk assessment framework [31, 32]. Predictor variables were selected based on three criteria: (i) established epidemiological evidence of their association with Aedes mosquito ecology and dengue transmission; (ii) availability of spatially continuous datasets covering the entire study area; and (iii) suitability for district-scale GIS analysis. Sixteen candidate predictors were screened; after redundancy screening (see below), 14 spatial predictor variables were retained and classified into three thematic domains: environmental suitability (6 variables), hazard (5 variables), and vulnerability (3 variables). The Environmental Suitability domain comprised climatic and land-surface variables, the Hazard domain included topographic and hydrological variables, and the Vulnerability domain represented population distribution and healthcare accessibility. Details of the predictor variables, data sources, spatial resolution, ecological rationale, and supporting references are summarized in Tables 1 and S18. Laboratory-confirmed dengue cases reported between January and December 2025 (*n* = 225) were obtained from the District Health Surveillance System and used for the spatial concordance assessment of the Dengue Risk Index (DRI). The single case reported under Gorakhpur Urban was assigned to Chargawan block, whose boundary contains Gorakhpur city, and three reporting units (Derwa, Karauta, and Ragar Ganj) reported no cases. No cases were recorded for Bharohiya in the 2025 line list, and the block was assigned zero cases; because the absence of reported cases cannot be distinguished from missing reports, a sensitivity analysis excluding Bharohiya was performed. Administrative block boundaries were used to integrate epidemiological records with the spatial predictor layers and to calculate block-level dengue incidence per 100,000 population. To avoid the temporal mismatch between 2025 case data and Census 2011 population counts, block populations for 2025 were obtained from the WorldPop Global2 constrained population estimates (release R2025A, version 1), which provide the estimated number of people per grid cell at a resolution of 3 arc-seconds (approximately 100 m at the equator) in the WGS84 geographic coordinate system [33]. These estimates are produced by random forest-based dasymetric redistribution of census-based population totals using remotely sensed and ancillary covariates, and are constrained to settled areas [34]. Block populations were obtained by summing grid-cell counts within each block boundary. To maintain temporal consistency, the most recent datasets corresponding to the study period were used whenever available. Satellite-derived variables were generated from Landsat-8/9 imagery; rainfall was calculated as the 2025 annual total (sum of daily values, mm) from the IMD 0.25° × 0.25° daily gridded rainfall dataset [28]; elevation and its derivatives were obtained from the Copernicus Global Digital Elevation Model (GLO-30, 30 m) distributed by OpenTopography [35]; and static datasets including the DEM, administrative boundaries, river and road networks, and healthcare facility locations were considered temporally invariant throughout the study period. Several potentially important determinants, including socioeconomic status, household water-storage practices, solid waste management, vector density, insecticide resistance, and detailed entomological surveillance data, were not incorporated, because spatially continuous district-level datasets were unavailable. Future studies should integrate these variables to enhance the comprehensiveness and accuracy of district-level spatial dengue risk assessment.

**Table 1:** Spatial predictor variables used for construction of the Integrated Dengue Risk Index (DRI)

Domain	Predictor variable	Data source	Acquisition year/date	Spatial resolution	Ecological/epidemiological rationale	References
Environmental suitability	Land Surface Temperature (LST)	Landsat-8/9 (USGS)	2025 (Annual mean)	30 m	Regulates mosquito development, survival, biting activity, and dengue virus replication	[3, 36, 37]
	Rainfall	IMD daily gridded rainfall (0.25° × 0.25°)	2025 (Annual total)	0.25° (~ 25 km)	Creates and replenishes mosquito breeding habitats	[28, 38]
	Normalized Difference Vegetation Index (NDVI)	Landsat-8/9	2025 (Annual mean)	30 m	Represents vegetation conditions influencing mosquito habitat suitability	[39, 40]
	Modified Normalized Difference Water Index (MNDWI)	Landsat-8/9	2025 (Annual mean)	30 m	Detects surface-water bodies and wet environments suitable for breeding	[39, 40]
	Normalized Difference Built-up Index (NDBI)	Landsat-8/9	2025 (Annual mean)	30 m	Represents urban built-up environments associated with *Aedes* habitats	[41]
	Land Use/Land Cover (LULC)	Landsat-8/9 (Random Forest classification)	2025 (post-monsoon composite)	30 m	Represents land-cover characteristics affecting vector habitat and human–vector interaction	[42, 43]
Hazard	Digital Elevation Model (DEM)	Copernicus DEM GLO-30 (OpenTopography)	2021 (Release)	30 m	Influences drainage, runoff, and local environmental conditions	[35, 41, 43]
	Slope	Derived from DEM	2025 (Derived)	30 m	Controls runoff and surface-water retention	[41]
	Aspect	Derived from DEM	2025 (Derived)	30 m	Influences solar radiation and surface moisture	[41, 43]
	Topographic Wetness Index (TWI)	Derived from DEM	2025 (Derived)	30 m	Represents potential soil moisture and water accumulation	[41]
	Distance to River	OpenStreetMap	2025 (Extracted)	30 m	Represents proximity to potential mosquito breeding habitats	[44, 45]
Vulnerability	Population Density	WorldPop Global2 constrained population estimates, R2025A v1 (people per grid cell; random forest-based dasymetric redistribution)	2025	3 arc-seconds (~ 100 m), summed to block	Represents the spatial concentration of the exposed population	[33, 34, 45]
	Household Density	Census of India	Census 2011	Block level	Represents residential clustering and human exposure	[15, 46]
	Distance to Health Facilities	OpenStreetMap/Health Department	2025 (Extracted)	30 m	Represents spatial accessibility to healthcare services	[47, 48]

Land Use/Land Cover (LULC) classification and accuracy assessment: LULC was classified into five classes: water, built-up, agriculture, vegetation, and open space. Landsat-8/9 Collection 2 Level-2 Surface Reflectance imagery was used to generate a cloud-masked post-monsoon composite for the study area. Clouds, cirrus, cloud shadows, and saturated pixels were removed using the QA_PIXEL and QA_RADSAT quality bands, followed by application of the Level-2 surface-reflectance scaling factors. The composite was generated using a median reducer, and spectral indices including NDVI, NDBI, NDWI, and BSI were incorporated as predictor variables. Reference LULC classes were derived from the reclassified GLC-FCS30D annual land-cover dataset, and edge pixels were reduced using a 3 × 3 local-mode filter to retain relatively homogeneous pixels. Stratified random sampling was performed at 30 m resolution with up to 1,000 samples per class. The resulting sample dataset was randomly divided into 70% training and 30% hold-out validation samples using a fixed random seed. A Random Forest classifier with 100 trees was trained using the Landsat spectral bands and derived indices. Classification performance was assessed using a confusion matrix, overall accuracy, kappa coefficient, producer’s accuracy, and user’s accuracy. The resulting accuracy assessment is reported in Supplementary Table S15. The five classes were then scored for Aedes habitat suitability as water = 5, built-up = 4, vegetation = 3, agriculture = 2, and open space = 1 (Supplementary Table S1a).

### Spatial data pre-processing and harmonization

All spatial datasets were pre-processed and harmonized to establish a consistent geospatial database for GIS-based multi-criteria analysis. Because the datasets originated from multiple sources with different coordinate systems, spatial resolutions, and formats, a standardized workflow was implemented to ensure spatial compatibility before analysis. All raster and vector datasets were projected to a common coordinate reference system (WGS 84/UTM Zone 44 N; EPSG:32644), clipped to the Gorakhpur district boundary, and harmonized to a 30 m × 30 m analysis grid. The 30 m grid represents the analytical resolution used for spatial overlay and index computation**,** rather than the effective spatial resolution of all input datasets. Landsat-8/9 imagery and the Copernicus DEM have a native spatial resolution of approximately 30 m, whereas other datasets had coarser native resolutions. Resampling to the 30 m analysis grid did not increase the effective spatial detail of coarser datasets. For example, IMD gridded rainfall (0.25°, approximately 25 km) was resampled by bilinear interpolation solely to achieve spatial compatibility and, therefore, represents regional-scale rainfall conditions rather than 30 m variability [28].

Continuous raster datasets were resampled using bilinear interpolation, whereas the categorical LULC layer was resampled using the nearest-neighbor method to preserve class integrity. Data quality was assessed by identifying missing values, No Data cells, and raster misalignment before analysis [49]. Topographic derivatives, including slope, aspect, and TWI, were generated from the Copernicus DEM, while Euclidean Distance analysis was used to derive continuous raster layers representing the distance to rivers, roads, and healthcare facilities [31]. All vector datasets were rasterized using the same projection, spatial extent, and 30 m cell size to ensure consistency during spatial overlay analysis [50]. Because the input layers were delivered on different grids (geographic 0.00027° grids and two Universal Transverse Mercator grids with sub-pixel offsets), all layers were resampled to a single reference grid before overlay. The resulting harmonized database comprised 14 georeferenced raster layers with identical coordinate systems, spatial extents, cell sizes, and raster alignment, providing a standardized analytical framework for subsequent variable standardization, weighting, and integration of the thematic indices used to construct the DRI.

### Standardization and reclassification of predictor variables

To enable integration of predictor variables measured in different units and ranges, all 14 predictor layers were standardized to a common five-level suitability scale before weighting and spatial analysis. Scores ranged from 1 (Very Low) to 5 (Very High), where higher scores represented conditions more favorable for Aedes mosquito survival, breeding, human exposure, and dengue transmission [27] (Table 2). The direction of each variable (positive or negative relationship with dengue suitability) was specified a priori from published evidence and the ecological requirements of Aedes mosquitoes (Supplementary Table S11): LST, rainfall, NDVI, MNDWI, NDBI, and TWI were scored positively, whereas elevation, slope, and distance to rivers were scored negatively (lower, flatter, and closer locations receiving higher scores). Continuous environmental and topographic variables were divided into five equal-interval classes between their observed district minimum and maximum [51]. Distance to rivers and distance to health facilities were classified into five 1.6 km and 1.2 km buffer bands, respectively; greater distance from a health facility was scored as greater vulnerability. Block-level population density was classified into five classes using Jenks Natural Breaks across the 20 blocks [52], and household density into five equal-interval classes. The LULC layer was reclassified based on the relative suitability of each land-cover class for Aedes habitats, with water bodies and built-up areas assigned higher suitability than less favorable land-cover types [51]. The complete reclassification criteria, suitability thresholds, and justification for all predictor variables are provided in Supplementary Table S1, with the full numeric/categorical reclassification thresholds underlying each 1–5 suitability score provided in Supplementary Table S1a. Standardization ensured analytical comparability among all predictor variables and provided the basis for subsequent AHP weighting and WLC integration.

**Table 2:** Standardized suitability scale used for reclassification of predictor variables

Suitability score	Risk category	Interpretation
1	Very low	Minimal contribution to dengue transmission potential
2	Low	Slight contribution to dengue transmission potential
3	Moderate	Moderate contribution to dengue transmission potential
4	High	High contribution to dengue transmission potential
5	Very high	Maximum contribution to dengue transmission potential

Aspect was treated as a circular directional variable rather than a linear continuous variable, recognizing that 0° and 360° represent the same north-facing direction. Accordingly, aspect was grouped into five directional sectors rather than assigning increasing suitability to increasing aspect degrees: north (337.5–22.5°) = 1, north–east and north–west (22.5–67.5° and 292.5–337.5°) = 2, east and west (67.5–112.5° and 247.5–292.5°) = 3, south–east and south–west (112.5–157.5° and 202.5–247.5°) = 4, and south (157.5–202.5°) = 5, reflecting the greater solar exposure and warmer surface conditions of south-facing slopes in the northern hemisphere, consistent with the positive scoring of LST. The north-facing sector was treated as continuous across the 0°/360° boundary (Supplementary Table S1a). Because the district is an almost flat alluvial plain (mean slope 1.35°), aspect carries little physical information and received the lowest weight in the Hazard Index; the scores represent relative suitability of aspect directions and do not imply a linear relationship between aspect degree and dengue risk.

### AHP weight assignment

The AHP was used to determine the relative importance of the predictor variables through pairwise comparisons within the MCDA framework [53–55]. Before weighting, candidate predictors were screened for redundancy using pixel-level Pearson and Spearman correlations (Supplementary Tables S14 and S25). Percent Vegetation was excluded because of strong collinearity with NDVI (*r* = 0.80). NDMI was excluded, because it was exactly the negative of NDBI (*r* = − 1.000), both being computed from the same near-infrared and shortwave-infrared bands; NDBI was retained, because built-up environments are a principal habitat determinant for Aedes aegypti [41]. The ERA5-Land relative humidity layer [56] was perfectly collinear (*r* = 1.000) with the rainfall layer initially prepared from the same reanalysis source, indicating that the two layers did not carry independent information; rainfall, which has the more direct and widely documented role in creating breeding habitats [38], was, therefore, re-derived from observed IMD gridded data [28], and relative humidity was excluded, because an independently derived layer of comparable quality was not available. The weights of the remaining six environmental criteria were derived from the corresponding 6 × 6 sub-matrix of the original expert consensus matrix, so that the expert judgements themselves were unchanged. The 14 retained predictor variables were grouped into three thematic domains: environmental suitability (6 variables), hazard (5 variables), and vulnerability (3 variables). An additional pairwise comparison was performed to determine the relative contributions of the three thematic indices (ESI, HI, and VI) in constructing the final DRI. Pairwise comparisons were conducted using Saaty's nine-point scale (1–9), where higher values indicate greater relative importance (Table 3).

**Table 3:** Saaty’s fundamental scale used for pairwise comparisons in AHP

Numerical value	Definition	Interpretation
1	Equal importance	Two criteria contribute equally to dengue transmission risk
3	Moderate importance	One criterion is moderately more important than the other
5	Strong importance	One criterion strongly influences dengue transmission risk
7	Very strong importance	One criterion has a very strong influence over another
9	Extreme importance	One criterion is overwhelmingly more important than another
2, 4, 6, 8	Intermediate values	Used when the importance lies between two adjacent judgments
1/2–1/9	Reciprocal values	Used when the compared criterion is less important than its counterpart

The pairwise comparison matrices were completed by a multidisciplinary panel of eight experts comprising public health specialists, an epidemiologist, a climatologist, an environmental scientist, a GIS and RS specialist, a medical geographer, and an entomologist. Experts independently completed the comparison matrices, after which a consensus matrix was established through discussion while maintaining logical consistency. The composition of the expert panel is provided in Supplementary Table S13. The normalized criterion weights satisfied [57]:

$$\sum_{i=1}^{n}{w}_{i}=1$$

where ${w}_{i}$ is the normalized weight of the *i*th criterion. Consistency of the pairwise comparisons was evaluated using Saaty's Consistency Index (CI) and consistency ratio (CR) [55, 58]:

$$\mathrm{C}\mathrm{I}=\frac{{\lambda}_{\mathrm{m}\mathrm{a}\mathrm{x}}-n}{n-1}$$

$${\mathrm{CR}} = \frac{{{\mathrm{CI}}}}{{{\mathrm{RI}}}}$$

where ${\lambda}_{\mathrm{m}\mathrm{a}\mathrm{x}}$ is the maximum eigenvalue, n is the matrix order, and $\mathrm{R}\mathrm{I}$ is the Random Index. Pairwise comparisons were considered acceptable when CR < 0.10. Weights were computed with the principal-eigenvector method. All comparison matrices satisfied this criterion, with CR values of 0.036 (Environmental Suitability), 0.050 (Hazard), 0.0079 (Vulnerability), and 0.0079 (DRI thematic), indicating satisfactory consistency (Table 4). Complete pairwise comparison matrices and the derived weights are provided in Supplementary Tables S2–S10. The normalized AHP weights were subsequently incorporated into the WLC approach to construct the ESI, HI, VI, and the final DRI.

**Table 4:** Consistency statistics for AHP comparison matrices

Comparison matrix	No. of Criteria	λmax	CI	RI	CR
Environmental Suitability	6	6.224	0.045	1.24	0.036
Hazard	5	5.225	0.056	1.12	0.050
Vulnerability	3	3.009	0.0046	0.58	0.0079
DRI thematic	3	3.009	0.0046	0.58	0.0079

All pairwise comparison matrices satisfied the acceptable consistency criterion (CR < 0.10), indicating satisfactory consistency

### Construction of the environmental suitability index (ESI)

The ESI was constructed by integrating six environmental variables representing climatic conditions, vegetation, surface water, urbanization, and land use (LST, rainfall, NDVI, MNDWI, NDBI, and LULC); Percent Vegetation, relative humidity, and NDMI were excluded following redundancy screening (Supplementary Table S14). The standardized raster layers were combined using the WLC approach with the normalized AHP weights [51, 58]. The ESI was calculated as

$$\mathrm{E}\mathrm{S}\mathrm{I}=\sum_{i=1}^{6}(\left.{W}_{i}\times {R}_{i}\right)$$

where ESI is the Environmental Suitability Index, ${W}_{i}$ is the normalized weight of the *i*th environmental variable, and ${R}_{i}$ is the corresponding standardized suitability score (1–5). The resulting ESI raster represents the relative spatial suitability of environmental conditions for *Aedes* mosquito survival and dengue transmission. The resulting ESI, therefore, lies on the same 1–5 scale as its inputs. The complete pairwise comparison matrix and normalized weights are provided in Supplementary Tables S2 and S3.

### Construction of the hazard index (HI)

The HI was constructed by integrating five topographic and hydrological variables representing terrain and surface-water conditions associated with dengue transmission. The standardized raster layers were combined using the WLC approach with the normalized AHP weights [58, 59]. The HI was calculated as

$$\mathrm{H}\mathrm{I}=\sum_{i=1}^{5}(\left.{W}_{i}\times {R}_{i}\right)$$

where HI is the Hazard Index, ${W}_{i}$ is the normalized weight of the *i*th hazard variable, and ${R}_{i}$ is the corresponding standardized suitability score (1–5). The resulting raster represents the relative spatial distribution of topographic and hydrological conditions associated with dengue transmission hazard. The complete pairwise comparison matrices and normalized weights are provided in Supplementary Tables S4 and S5.

### Construction of the vulnerability index (VI)

The VI was constructed by integrating three socio-demographic variables (Population Density, Household Density, and Distance to Health Facilities) representing population exposure and healthcare accessibility. Population density was calculated for each block as the 2025 WorldPop population divided by block area [33], and household density was obtained from Census 2011. The standardized raster layers were combined using the WLC approach with the normalized AHP weights [55, 58]. The VI was calculated as

$$\mathrm{V}\mathrm{I}=\sum_{i=1}^{3}(\left.{W}_{i}\times {R}_{i}\right)$$

where VI is the Vulnerability Index, ${W}_{i}$ is the normalized weight of the *i*th vulnerability variable, and ${R}_{i}$ is the corresponding standardized suitability score (1–5). The resulting raster represents the relative spatial distribution of population vulnerability to dengue transmission. The complete pairwise comparison matrices and normalized weights are provided in Supplementary Tables S6 and S7.

### Construction of the Integrated Dengue Risk Index (DRI)

The DRI was constructed by integrating the ESI, HI, and VI using the WLC approach. The relative contributions of the three thematic indices were determined from the normalized AHP weights [27, 55] (Table 5). The DRI was calculated as

$$\mathrm{D}\mathrm{R}\mathrm{I}=\left({W}_{\mathrm{E}\mathrm{S}\mathrm{I}}\times \mathrm{E}\mathrm{S}\mathrm{I}\right)+\left({W}_{\mathrm{H}\mathrm{I}}\times \mathrm{H}\mathrm{I}\right)+\left({W}_{\mathrm{V}\mathrm{I}}\times \mathrm{V}\mathrm{I}\right)$$

where ${W}_{\mathrm{E}\mathrm{S}\mathrm{I}}$, ${W}_{\mathrm{H}\mathrm{I}}$, and ${W}_{\mathrm{V}\mathrm{I}}$ are the normalized weights assigned to the ESI, HI, and VI, respectively. Substituting the normalized thematic weights, the final equation was

**Table 5:** Final AHP weights assigned to the thematic indices used to construct the DRI

Thematic Index	Weight
Environmental Suitability Index (ESI)	0.540
Vulnerability Index (VI)	0.297
Hazard Index (HI)	0.163

$$\mathrm{D}\mathrm{R}\mathrm{I}=\left(0.540\times \mathrm{E}\mathrm{S}\mathrm{I}\right)+\left(0.163\times \mathrm{H}\mathrm{I}\right)+\left(0.297\times \mathrm{V}\mathrm{I}\right)$$

Because each thematic index is a weighted mean of 1–5 scores and the thematic weights sum to one, the DRI is also expressed on a 1 (lowest) to 5 (highest) relative scale. DRI values are unitless composite scores that rank locations within the district; they have no direct epidemiological interpretation (for example, as an expected incidence). The continuous DRI surface was classified into five risk categories (Very Low, Low, Moderate, High, and Very High) using the Jenks Natural Breaks method [52] to facilitate interpretation and visualization. Because Jenks breaks are data-dependent, these classes are relative to the distribution of DRI values in Gorakhpur district and should not be interpreted as epidemiologically calibrated, absolute risk categories. The complete thematic pairwise comparison matrix and normalized weights are provided in Supplementary Tables S8–S10.

### Statistical analysis and spatial concordance assessment

Spatial concordance between the DRI and observed dengue incidence was assessed by comparing block-level mean DRI values with observed dengue incidence across the 20 administrative blocks of Gorakhpur district during 2025. Mean DRI values were calculated for each block by zonal statistics and linked with laboratory-confirmed dengue incidence (cases per 100,000 population, using WorldPop 2025 block populations as denominators). The relationship between mean DRI and observed incidence was evaluated using Pearson's product-moment correlation coefficient (*r*) and simple linear regression [60–62]. Because the analysis was based on only 20 blocks and incidence may not be normally distributed, Spearman's rank correlation (*ρ*) and Kendall's *τ* were additionally calculated, and a 95% bootstrap confidence interval for r was obtained from 10,000 resamples of blocks. Normality of incidence and of regression residuals was tested with the Shapiro–Wilk test, homoscedasticity with the Breusch–Pagan test, and the influence of individual blocks with Cook's distance and leave-one-out correlations [63]. Because case counts were overdispersed relative to a Poisson distribution, a negative binomial regression of case counts on mean DRI with log(population) as an offset was fitted as a count-based sensitivity analysis and summarized as the incidence rate ratio (IRR) per 0.1-unit increase in DRI [64]. Pearson's correlation coefficient was calculated as

$$r=\frac{\sum_{i=1}^{n}(\left.{x}_{i}-\overline{x }\right)\left({y}_{i}-\overline{y }\right)}{\sqrt{\sum_{i=1}^{n}({x}_{i}-\overline{x }{)}^{2}}\sqrt{\sum_{i=1}^{n}({y}_{i}-\overline{y }{)}^{2}}}$$

where ${x}_{i}$ and ${y}_{i}$ represent the mean DRI and observed dengue incidence for the *i*th administrative block, respectively. The regression model was expressed as

$$Y={\beta}_{0}+{\beta}_{1}X+\varepsilon$$

where Y is the observed dengue incidence, X is the mean DRI, ${\beta}_{0}$ is the intercept, ${\beta}_{1}$ is the regression coefficient, and ε is the random error. Model agreement was evaluated using the coefficient of determination (R^2^), RMSE, and MAE [65, 66]:

$${R}^{2}=1-\frac{\sum_{i=1}^{n}({y}_{i}-{\widehat{y}}_{i}{)}^{2}}{\sum_{i=1}^{n}({y}_{i}-\overline{y }{)}^{2}}$$

$$\mathrm{R}\mathrm{M}\mathrm{S}\mathrm{E}=\sqrt{\frac{1}{n}\sum_{i=1}^{n}({y}_{i}-{\widehat{y}}_{i}{)}^{2}}$$

$$\mathrm{M}\mathrm{A}\mathrm{E}=\frac{1}{n}\sum_{i=1}^{n}\mid {y}_{i}-{\widehat{y}}_{i}\mid$$

Spatial dependence was assessed with global Moran’s I [67] for block-level DRI, incidence, and OLS residuals, using a row-standardized queen-contiguity weights matrix (with a 60 m snapping tolerance to close digitizing gaps between adjoining block polygons) and, as a robustness check, a four-nearest-neighbor matrix; significance was assessed by 9,999 random permutations. Lagrange multiplier tests for spatial error and spatial lag dependence were applied to the OLS residuals [68]. A spatial error or spatial lag model was to be fitted if residual spatial dependence was significant. Sensitivity analyses repeated the concordance assessment excluding the most influential block, excluding Bharohiya (no reported cases), and using Census 2011 denominators, and recomputed the DRI with rainfall restricted to the June–October transmission season. A scatter plot with a fitted regression line was used to visualize the relationship between mean DRI and observed incidence (Supplementary Figure S1). As the objective of the framework was to describe relative spatial susceptibility rather than to predict disease incidence, these statistics were interpreted as measures of spatial concordance rather than predictive performance. Spatial processing was performed in QGIS 3.44; raster harmonization, index construction, zonal statistics, and statistical analyses were performed in Python 3.11 using NumPy 2.4, SciPy 1.17, pandas 3.0, tifffile, OpenCV, and Matplotlib 3.10, with custom routines for Jenks classification, Moran's I, and Lagrange multiplier tests. Two-sided *p* < 0.05 was considered statistically significant.

## Results

### Spatial distribution of environmental determinants associated with dengue transmission

**Fig. 3:**
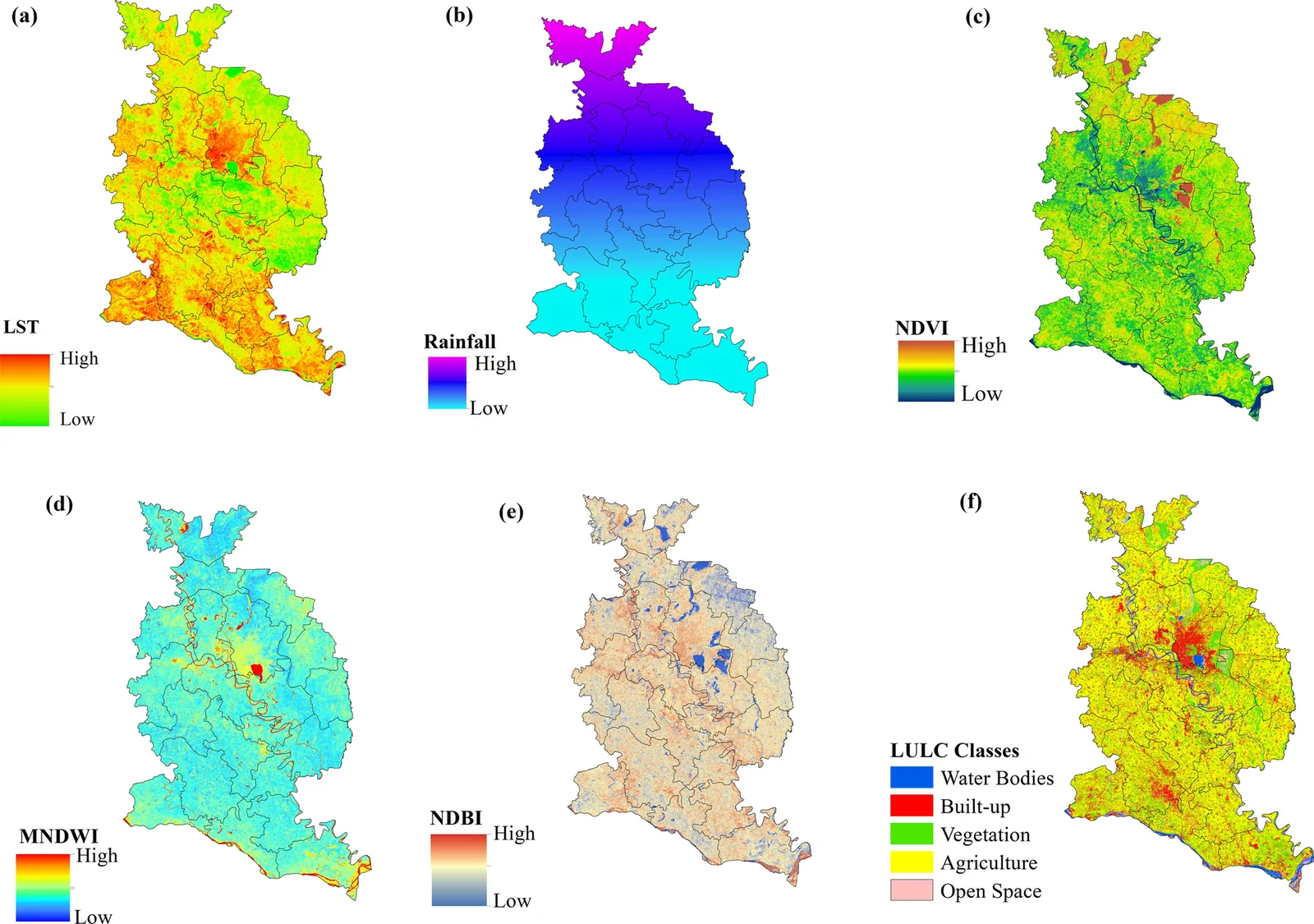
Spatial distribution of environmental determinants used for dengue risk assessment in Gorakhpur district. Spatial distribution of the environmental variables used to construct the DRI: **a** LST (2025 annual mean), **b** Rainfall (2025 annual total, IMD 0.25° gridded data), **c** NDVI, **d** MNDWI, **e** NDBI, and **f** LULC. Relative humidity and NDMI were excluded after redundancy screening (see Methods).

Environmental determinants showed clear spatial heterogeneity across Gorakhpur district (Fig. 3). Across the district, annual mean LST ranged from 22.96 to 45.60 °C (mean 32.69 ± 2.30 °C), with the highest values in Gorakhpur city and adjoining built-up areas (Fig. 3a). Total rainfall in 2025 ranged from 277 to 1,033 mm (mean 689 ± 203 mm) and showed a west–east gradient, with the highest totals in the western and north-western blocks and the lowest in the east-central part of the district (Fig. 3b); because the IMD grid has a spacing of 0.25°, this layer reflects regional rather than local variation. Vegetation patterns differed spatially, with NDVI ranging from −0.26 to 0.86 (mean 0.43 ± 0.11) and higher values predominantly occurring in peripheral and southern rural areas, while lower values occurred around the urban core (Fig. 3c). MNDWI ranged from −0.56 to 0.69 (− 0.36 ± 0.11), reflecting localized surface-water conditions (Fig. 3d). NDBI ranged from −0.78 to 0.29 (− 0.09 ± 0.09), with higher values concentrated in Gorakhpur city and adjacent peri-urban areas, contrasting with the predominantly agricultural landscape elsewhere (Fig. 3e). LULC further demonstrated the heterogeneous mixture of built-up areas, agriculture, vegetation, water bodies, and open land (Fig. 3f). Complete summary statistics and observed ranges for the environmental predictors are provided in Supplementary Tables S19 and S25.

Redundancy screening identified two pairs of perfectly collinear candidate layers: the ERA5-land relative humidity layer and the rainfall layer initially prepared from the same source (pixel-level Pearson’s *r* = 1.000), and NDMI and NDBI (*r* = −1.000), which are algebraic negatives of each other. Relative humidity and NDMI were, therefore, removed, and rainfall was re-derived from IMD observed gridded data, which correlated only moderately with the relative humidity layer (*r* = 0.47). Among the six retained environmental predictors, the strongest remaining correlations were between MNDWI and NDBI (*r* = −0.52), NDVI and NDBI (*r* = −0.47), and LST and NDBI (*r* = 0.42); rainfall was essentially uncorrelated with the spectral predictors (|r|≤ 0.07) (Supplementary Tables S14 and S25).

The spatial co-occurrence of higher rainfall in the west and of built-up, warmer surfaces around Gorakhpur city suggests that environmental conditions favorable to dengue transmission suitability arise from different factors in different parts of the district. Conversely, the comparatively lower rainfall in the east-central blocks and the more rural landscape in the south–east contribute to lower environmental suitability in those areas.

### Spatial distribution of hazard and vulnerability determinants

**Fig. 4:**
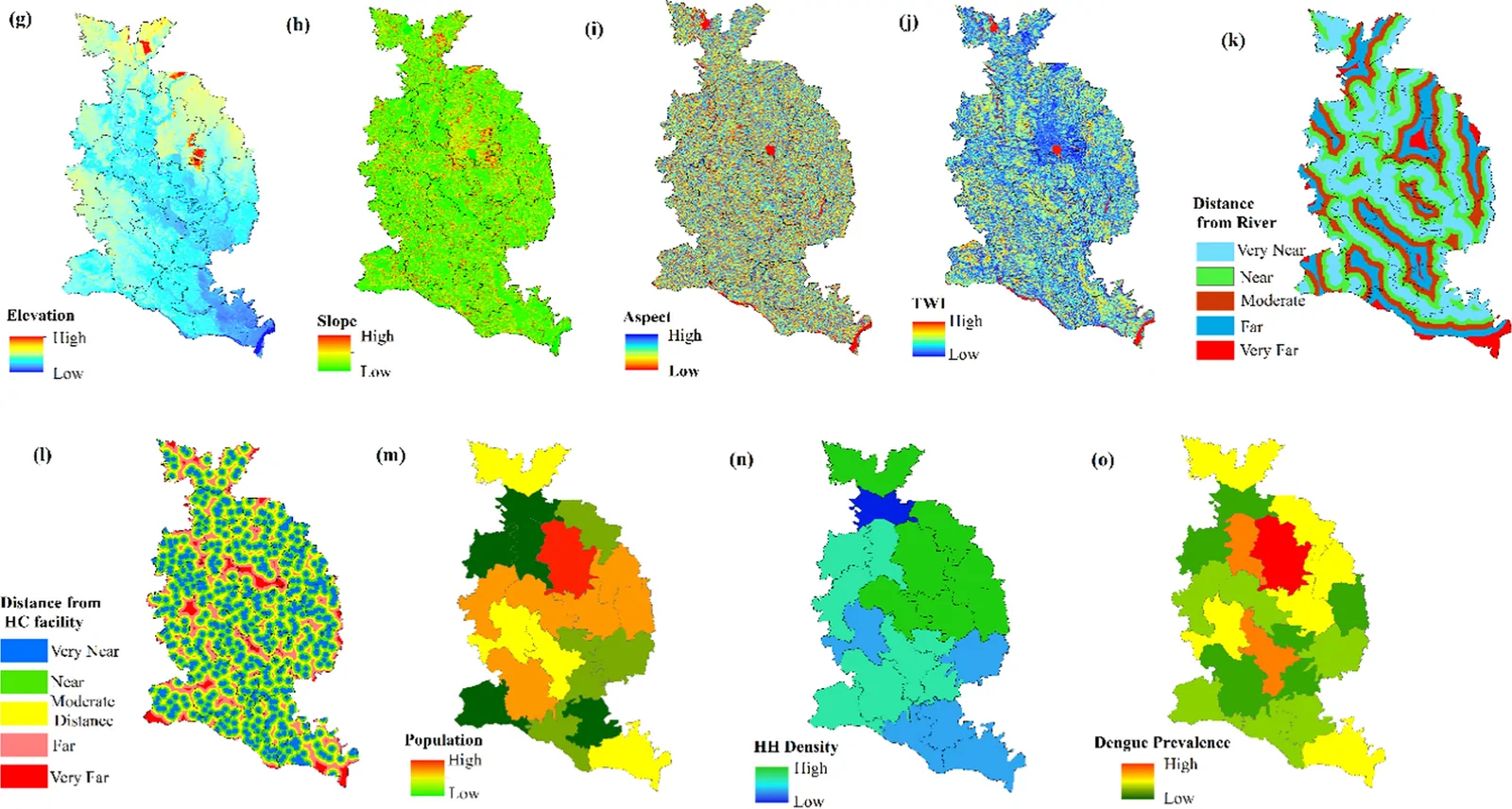
Spatial distribution of hazard and vulnerability determinants used for dengue risk assessment in Gorakhpur district. Spatial distribution of the hazard and vulnerability variables used to construct the HI, VI, and DRI: **a** elevation (Copernicus DEM), **b** slope, **c** aspect sector, **d** TWI, **e** distance to river, **f** distance to healthcare facilities, **g** population density (WorldPop 2025), **h** household density (Census 2011), and **i** Reported Dengue Incidence, 2025 (per 100,000 population)

Hazard and vulnerability variables also showed distinct spatial patterns across Gorakhpur district (Fig. 4). Elevation decreased from the north–west toward the south–eastern floodplain, while slope remained generally low and aspect showed no dominant spatial pattern (Fig. 4a–c). Across the 20 administrative blocks, mean elevation ranged from 69.47 to 82.50 m (mean 76.84 ± 3.22 m), mean slope from 1.09 to 1.88° (1.34 ± 0.20°), and mean TWI from 0.41 to 0.47 (0.44 ± 0.01). TWI identified localized areas of greater surface wetness associated with low-lying terrain and drainage corridors, while distance to rivers reflected proximity gradients around the major river systems (Fig. 4d, e). At the pixel level, TWI and slope were negatively correlated (Pearson's *r* = −0.73), and elevation was weakly correlated with slope (*r* = 0.29) and TWI (*r* = −0.30), indicating some shared topographic structure; all correlations among the standardized hazard scores were ≤ 0.23 in absolute value (Supplementary Table S27).

Healthcare accessibility varied across administrative blocks, while population and household densities were concentrated in central urban and peri-urban areas and declined toward the rural periphery (Fig. 4f–h). Population density in 2025 ranged from 1,058 persons/km^2^ in Campierganj to 8,135 persons/km^2^ in Chargawan, which contains Gorakhpur city (median 1,450; mean 1,889 ± 1,520), while household density ranged from 99.48 to 228.60 households/km^2^. Distance to healthcare facilities ranged from 0 to 6 km. At the block level, population and household density were moderately correlated (Pearson’s *r* = 0.54), whereas healthcare accessibility showed weak correlations with population density (*r* = 0.17) and household density (*r* = 0.02), suggesting that population concentration and healthcare accessibility represent relatively distinct dimensions of vulnerability (Supplementary Table S26).

Reported dengue incidence in 2025 ranged from 0 (Sardarnagar and Bharohiya) to 7.17 per 100,000 in Jungle Kaudia, with higher values also in Bansgaon (6.87), Chargawan (5.68), and Bhathat (5.29) (Fig. 4i; Table 8). High-incidence blocks, therefore, occurred both in the north-central part of the district and in Bansgaon in the south-center. The relatively localized variation in topographic and hydrological conditions, together with the concentration of population and households in central and peri-urban areas, indicates that environmental hazard and population vulnerability vary through distinct spatial mechanisms, which provides the rationale for integrating these components into the HI, VI, and final DRI rather than interpreting any single determinant in isolation.

### Environmental Suitability Index (ESI)

**Fig. 5:**
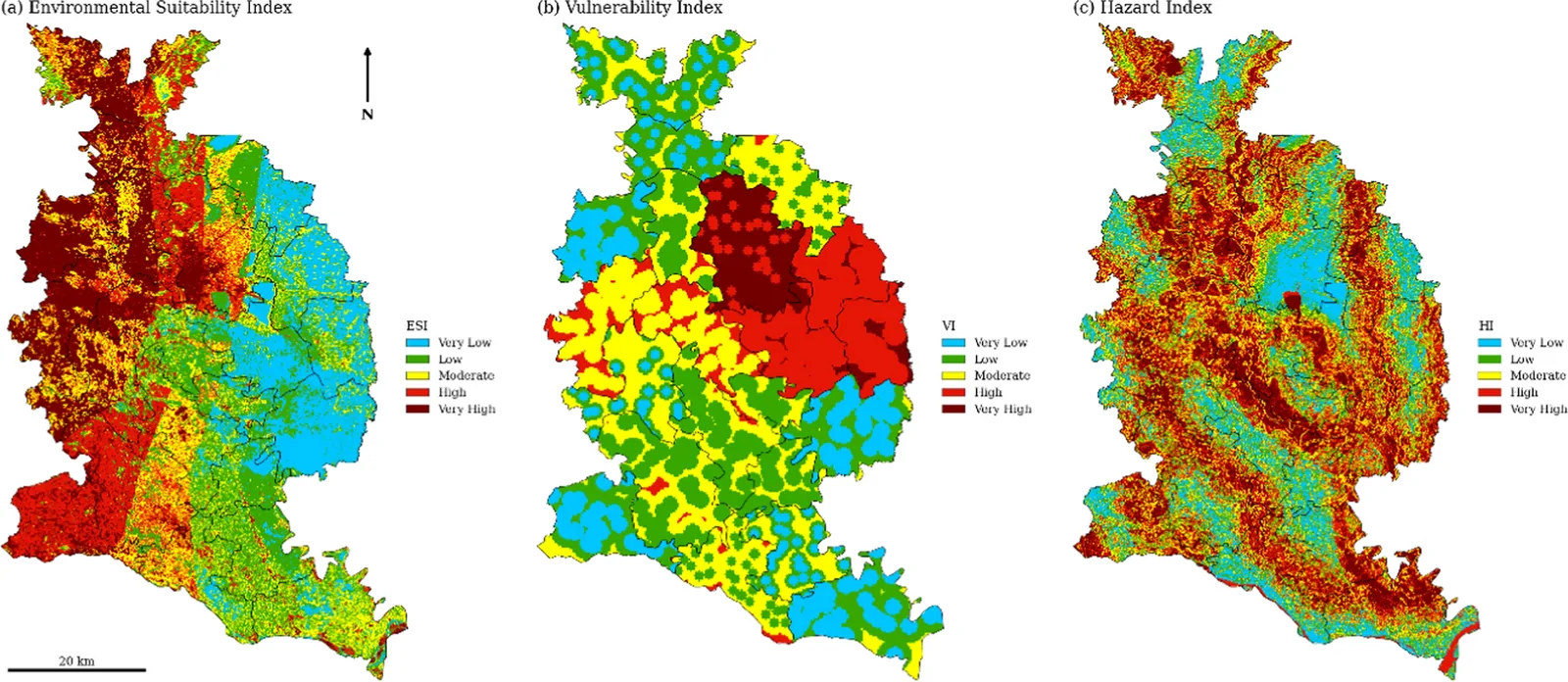
Spatial distribution of the composite indices used for dengue risk assessment. Spatial distribution of the composite indices generated using AHP: **a** ESI, **b** VI, and **c** HI. Each index is classified into five relative categories (Very Low, Low, Moderate, High, and Very High) using Jenks Natural Breaks computed separately for each index

The ESI showed pronounced spatial heterogeneity across Gorakhpur district, with a west–east gradient in dengue transmission suitability (Fig. 5a). Block mean ESI values ranged from 2.06 in Brahmpur to 3.24 in Pali on the 1–5 scale (Table 6; Supplementary Table S24). Higher ESI values were concentrated in the western blocks, particularly Pali, Sahjanwa, Jungle Kaudia, Belghat, Bharohiya, and Khajni, whereas the eastern blocks (Brahmpur, Khorabar, Sardarnagar, Pipraich, and Bhathat) showed the lowest suitability.

**Table 6:** Mean values of the ESI, VI, HI, and DRI across the administrative blocks of Gorakhpur district (all indices on a 1–5 relative scale)

Rank	Administrative block	ESI	VI	HI	DRI	Risk class
1	Chargawan	2.921	4.284	3.075	3.351	Very high
2	Sahjanwa	3.186	2.725	3.404	3.085	High
3	Jungle Kaudia	3.154	2.306	3.702	2.992	High
4	Piprauli	2.845	2.894	3.625	2.987	High
5	Sardarnagar	2.322	3.643	3.313	2.877	Moderate
6	Khajni	3.050	2.216	3.502	2.876	Moderate
7	Uruwa	2.970	2.273	3.423	2.837	Moderate
8	Pali	3.236	1.709	3.468	2.820	Moderate
9	Pipraich	2.342	3.418	3.277	2.815	Moderate
10	Belghat	3.118	1.803	3.455	2.783	Moderate
11	Khorabar	2.208	3.452	3.432	2.778	Moderate
12	Campierganj	3.017	1.963	3.335	2.757	Moderate
13	Bharohiya	3.059	1.911	3.261	2.751	Moderate
14	Gola	2.667	2.539	3.229	2.721	Moderate
15	Bansgaon	2.697	2.229	3.486	2.687	Moderate
16	Gagaha	2.553	2.045	3.494	2.556	Low
17	Bhathat	2.377	2.351	3.358	2.530	Low
18	Kauriram	2.426	2.208	3.372	2.516	Low
19	Barhalganj	2.597	1.632	3.454	2.450	Low
20	Brahmpur	2.060	1.564	3.661	2.174	Very low

This spatial pattern indicates that favorable environmental conditions were clustered rather than uniformly distributed across the district (Moran's I for block mean ESI = 0.60, *p* < 0.001; Supplementary Table S28). The pattern reflects the combined spatial configuration of the six environmental predictors used in the ESI (LST, rainfall, NDVI, MNDWI, NDBI, and LULC), which were standardized to a common 1–5 suitability scale before AHP weighting (Supplementary Tables S1–S3). Among these predictors, LST (AHP weight = 0.463) and rainfall (0.241) had the greatest influence on the ESI, followed by LULC (0.125). The west–east ESI gradient, therefore, largely follows the 2025 rainfall distribution, whereas higher LST and built-up land cover raise suitability locally around Gorakhpur city. Because rainfall was available only on a 0.25° grid, part of this gradient reflects regional rather than local differences in conditions.

### Vulnerability Index (VI)

The VI showed substantial spatial heterogeneity across Gorakhpur district, with block means ranging from 1.56 in Brahmpur to 4.28 in Chargawan (Fig. 5b; Table 6; Supplementary Table S24). Higher vulnerability was concentrated mainly in the central blocks, particularly Chargawan, Sardarnagar, Khorabar, and Pipraich, whereas Brahmpur, Barhalganj, Pali, Belghat, and Bharohiya showed comparatively lower vulnerability. This pattern indicates an uneven spatial distribution of population vulnerability (Moran’s *I* = 0.26, *p* = 0.03). Notably, Pali and Bharohiya showed high environmental suitability but comparatively low vulnerability, demonstrating that environmental suitability and population vulnerability represent distinct spatial dimensions of dengue susceptibility (Table 6).

### Hazard Index (HI)

Compared with the ESI and VI, the HI showed relatively limited spatial variability between blocks, with mean values ranging from 3.08 in Chargawan to 3.70 in Jungle Kaudia (Fig. 5c; Table 6; Supplementary Table S24), and no significant spatial autocorrelation (Moran's *I* = 0.03, *p* = 0.59). Within blocks, HI varied along drainage corridors and low-lying floodplain areas. The relatively narrow between-block range indicates that hazard-related conditions were more spatially homogeneous at block scale than environmental suitability and vulnerability, suggesting that differences in overall DRI were driven more strongly by variation in ESI and VI than by HI (Table 6).

Table 6 summarizes the block mean values of the ESI, VI, HI, and DRI, facilitating comparison of the relative contributions of environmental suitability, vulnerability, and hazard to the spatial distribution of dengue susceptibility across Gorakhpur district. All four indices are weighted means of 1–5 suitability scores and are, therefore, expressed on the same 1 (lowest) to 5 (highest) relative scale; the values are unitless and do not have a direct epidemiological meaning. The risk class of each block was assigned by applying the district-wide Jenks breaks of the DRI raster (2.351, 2.647, 2.905, and 3.212) to the block mean.

### Integrated Dengue Risk Index (DRI)

**Fig. 6:**
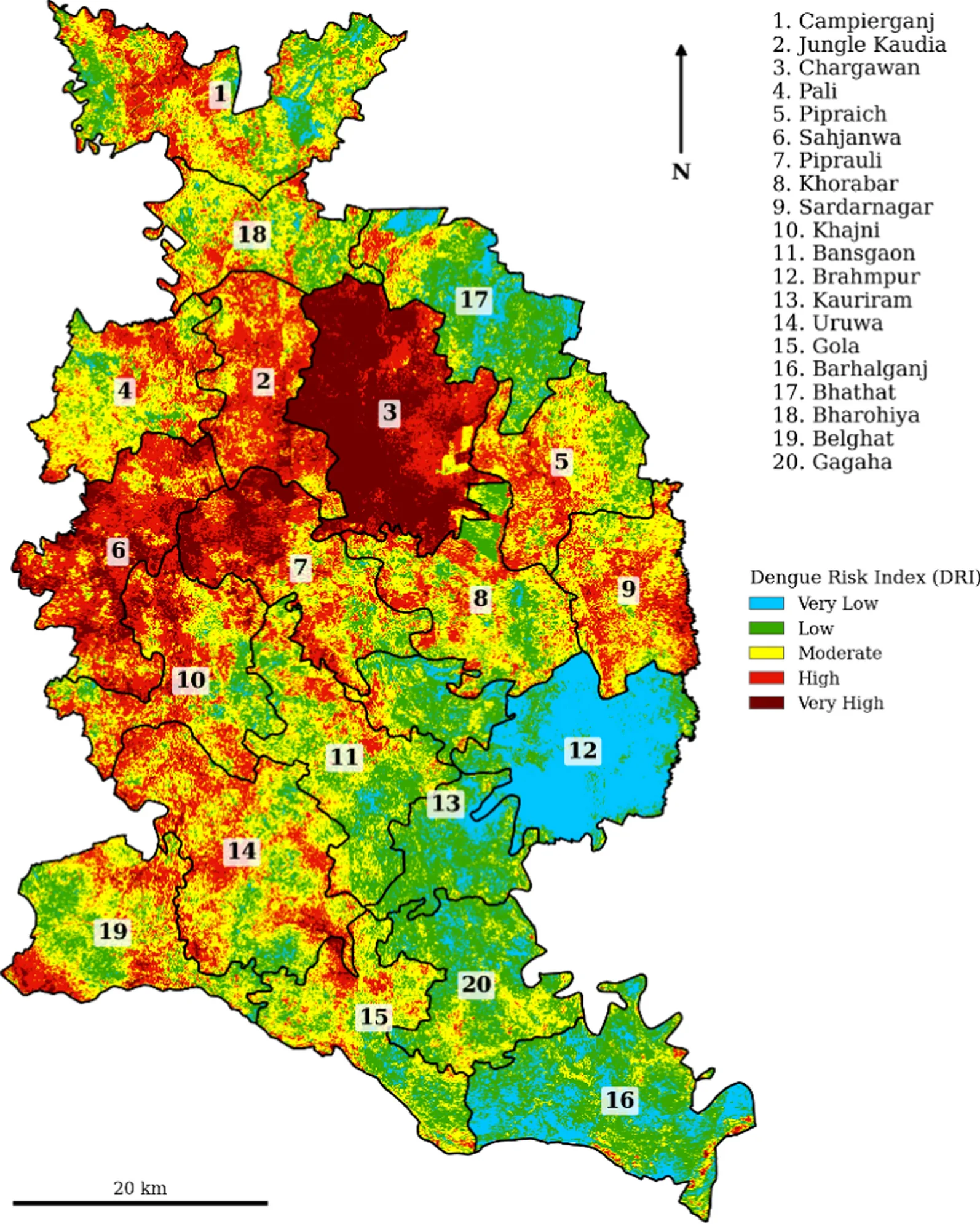
Spatial distribution of the DRI across Gorakhpur district (block numbers as listed)

The DRI showed marked spatial heterogeneity across Gorakhpur district, with pixel values ranging from 1.42 to 4.28 and block means ranging from 2.17 in Brahmpur to 3.35 in Chargawan (Fig. 6; Table 6; Supplementary Table S21). Higher DRI values were concentrated in Chargawan and in the central-western blocks, whereas lower values occurred in the eastern and south-eastern blocks. Block mean DRI showed significant positive spatial autocorrelation with both queen contiguity (Moran’s *I* = 0.22, *p* = 0.048) and four-nearest-neighbor weights (*I* = 0.32, *p* = 0.003). Chargawan was the only block classified as Very High; Sahjanwa, Piprauli, and Jungle Kaudia were classified as High; and Brahmpur as Very Low. This pattern indicates that the DRI emerged from the combined spatial distribution of environmental suitability, population vulnerability, and hazard conditions, rather than from a single thematic component.

The distribution of higher DRI values is consistent with the greater contribution of ESI (weight = 0.540) and VI (weight = 0.297) compared with HI (weight = 0.163) (Supplementary Table S9). Thus, spatial differences in environmental suitability and population vulnerability contributed more strongly to the differentiation of overall susceptibility than hazard alone. Chargawan ranked highest because of very high vulnerability combined with moderately high environmental suitability, whereas Sahjanwa and Jungle Kaudia ranked highly mainly because of high environmental suitability; conversely, Sardarnagar and Pipraich had high vulnerability but low environmental suitability and were classified as Moderate. These contrasts illustrate the value of integrating the three thematic dimensions (Table 6; Supplementary Table S24).

The DRI was generated by weighted integration of ESI, HI, and VI using the AHP–WLC framework and classified into Very Low, Low, Moderate, High, and Very High categories using Jenks Natural Breaks (Supplementary Tables S9 and S10). Classes are relative to this district. Block rankings were stable under equal thematic weights and ± 20% perturbation of each thematic weight (Spearman's ρ with the baseline ranking 0.93–1.00; 4–5 of the five highest-ranked blocks retained), under ± 10% perturbation of each individual criterion weight (ρ ≥ 0.99), and when rainfall was restricted to the June–October season (*ρ* = 0.99) (Supplementary Tables S16 and S17).

### Spatial extent of the DRI

**Fig. 7:**
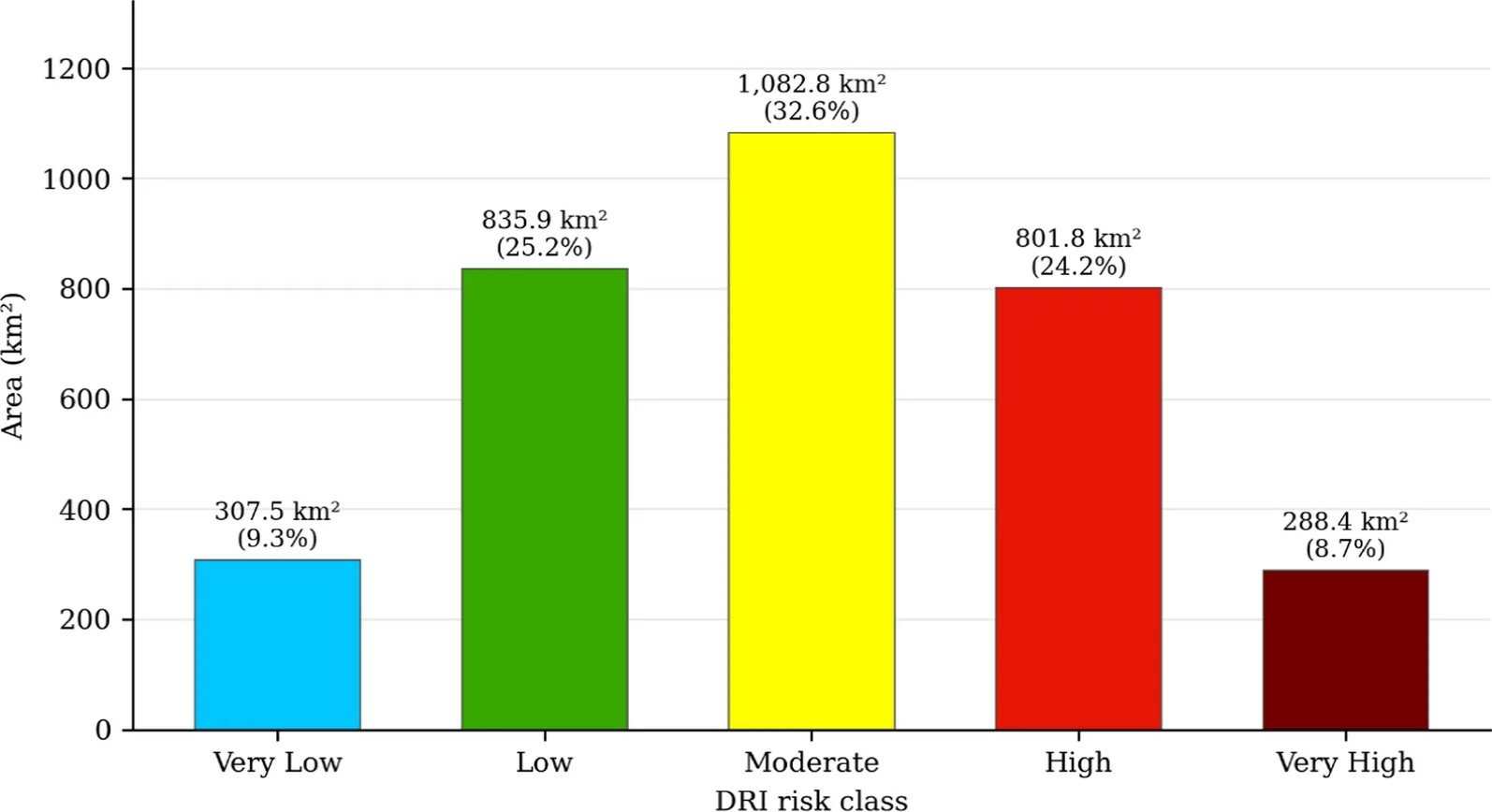
Area of DRI risk categories across Gorakhpur district. Area (km^2^) and percentage of district area in the five DRI risk categories (Very Low, Low, Moderate, High, and Very High), based on 30 m pixel counts within the 20 block boundaries

The DRI classified Gorakhpur district into five relative risk categories, with the Moderate class occupying the largest area (1,082.8 km^2^; 32.65%), followed by the Low (835.9 km^2^; 25.21%), High (801.8 km^2^; 24.18%), Very Low (307.5 km^2^; 9.27%), and Very High (288.4 km^2^; 8.70%) classes (Fig. 7). Overall, 32.87% of the district (1,090.2 km^2^) was classified as High or Very High, whereas 34.48% (1,143.4 km^2^) fell within the Low or Very Low categories. High and Very High areas were spatially clustered in and around Gorakhpur city and in the central-western blocks rather than uniformly distributed. Because the classes are defined by data-dependent Jenks breaks, these areas describe relative differences within the district rather than absolute levels of transmission risk (Fig. 7; Table 6; Supplementary Table S21).

### Spatial concordance between the DRI and observed dengue incidence

Spatial concordance between the DRI and observed dengue incidence was assessed by comparing the mean DRI of each administrative block with the corresponding observed incidence in 2025 (225 laboratory-confirmed cases). The association was weak and not statistically significant (Pearson’s *r* = 0.164, *p* = 0.491; bootstrap 95% CI − 0.41 to 0.53) (Table 7). Rank-based measures showed no monotonic agreement (Spearman’s *ρ* = −0.003, *p* = 0.990; Kendall’s *τ* = −0.026). Block incidence did not depart significantly from normality (Shapiro–Wilk *W* = 0.906, *p* = 0.053), supporting the use of Pearson's correlation alongside the rank-based measures. Simple linear regression gave the following equation:

**Table 7:** Statistical measures of spatial concordance between the block mean DRI and observed dengue incidence, 2025 (*n* = 20 blocks)

Parameter	Value
Pearson correlation coefficient (r)	0.164
*p* value	0.491
Bootstrap 95% CI for *r* (10,000 resamples)	− 0.41 to 0.53
Spearman's rank correlation (ρ)	− 0.003
Spearman's *p* value	0.990
Kendall's rank correlation (τ)	− 0.026
Regression equation	Incidence = − 1.301 + 1.427 × Mean DRI
Regression slope	1.427
Intercept	− 1.301
Coefficient of determination (*R*^2^)	0.027
Root Mean Square Error (RMSE)	2.107
Mean Absolute Error (MAE)	1.758
Shapiro–Wilk test of residuals	*W* = 0.929, *p* = 0.149
Breusch–Pagan test	*χ*^2^ = 0.76, *p* = 0.382
Maximum Cook's distance	0.37 (Chargawan)
Negative binomial IRR per 0.1 DRI (95% CI)	1.05 (0.91–1.21), *p* = 0.498
Moran's I, incidence (queen)	− 0.125 (*p* = 0.626)
Moran's I, OLS residuals (queen)	− 0.166 (*p* = 0.444)

DengueIncidence=-1.301+1.427×MeanDRI

The regression explained 2.7% of the between-block variation in incidence (*R*^2^ = 0.027; RMSE = 2.107; MAE = 1.758) (Table 7). Regression assumptions were acceptable: residuals did not depart from normality (Shapiro–Wilk *W* = 0.929, *p* = 0.149) or homoscedasticity (Breusch–Pagan χ^2^ = 0.76, *p* = 0.382), and no block was unduly influential (maximum Cook's distance 0.37, Chargawan). Because case counts were overdispersed (Poisson dispersion ratio 4.59), a negative binomial model with a population offset was fitted; it gave a similar, non-significant result (IRR per 0.1-unit increase in DRI = 1.05, 95% CI 0.91–1.21, *p* = 0.498). Neither incidence (Moran’s *I* = − 0.13, *p* = 0.63) nor the OLS residuals (Moran's *I* = − 0.17, *p* = 0.44; Lagrange multiplier error = 1.03, *p* = 0.31; lag = 0.81, *p* = 0.37) showed significant spatial autocorrelation with queen contiguity, and results were similar with four-nearest-neighbor weights (Supplementary Table S28); spatial regression was, therefore, not required and OLS inference was retained. In sensitivity analyses, excluding Chargawan (*r* = − 0.019, *p* = 0.940) or Bharohiya (*r* = 0.166, *p* = 0.497) did not change the conclusion, whereas using Census 2011 denominators produced an apparently significant Pearson correlation (*r* = 0.524, *p* = 0.021) that was driven entirely by Chargawan (Cook's distance 4.04; Spearman's *ρ* = − 0.005; *r* = − 0.065 without Chargawan), because the Census 2011 figure for Chargawan excludes the population of Gorakhpur city contained within its boundary (Supplementary Tables S22 and S23).

Observed and regression-estimated incidence differed substantially among blocks (Table 8). The largest positive residuals occurred in Bansgaon (+ 4.33), Jungle Kaudia (+ 4.20), and Bhathat (+ 2.98), where observed incidence exceeded the value expected from the DRI, whereas the largest negative residuals occurred in Sardarnagar (− 2.80) and Bharohiya (− 2.62), which reported no cases despite Moderate DRI, and in Uruwa (− 2.45) and Sahjanwa (− 2.15). When blocks were grouped by DRI class, the single very high-risk block (Chargawan) accounted for 110 of the 225 cases (48.9%) and had a pooled incidence of 5.68 per 100,000, compared with 2.54, 2.27, 3.05, and 1.28 per 100,000 in the High, Moderate, Low, and Very Low classes; incidence did not differ significantly across classes (Kruskal–Wallis H = 2.75, *p* = 0.60; Supplementary Table S29). Overall, the spatial concordance between the DRI and single-year block-level incidence was weak and not statistically significant, and the DRI should not be regarded as validated against dengue incidence.

**Table 8:** Observed and regression-estimated dengue incidence across administrative blocks, 2025 (incidence per 100,000 population, WorldPop 2025 denominators)

S. No	Block	Mean DRI	Observed incidence (per 100,000)	Regression-estimated incidence	Residual
1	Campierganj	2.757	3.27	2.63	0.64
2	Jungle Kaudia	2.992	7.17	2.97	4.20
3	Chargawan	3.351	5.68	3.48	2.20
4	Pali	2.820	1.28	2.72	− 1.44
5	Pipraich	2.815	3.65	2.72	0.94
6	Sahjanwa	3.085	0.96	3.10	− 2.15
7	Piprauli	2.987	1.37	2.96	− 1.59
8	Khorabar	2.778	2.32	2.66	− 0.35
9	Sardarnagar	2.877	0.00	2.80	− 2.80
10	Khajni	2.876	3.50	2.80	0.69
11	Bansgaon	2.687	6.87	2.53	4.33
12	Brahmpur	2.174	1.28	1.80	− 0.53
13	Kauriram	2.516	0.87	2.29	− 1.42
14	Uruwa	2.837	0.30	2.75	− 2.45
15	Gola	2.721	1.70	2.58	−0.89
16	Barhalganj	2.450	3.79	2.20	1.59
17	Bhathat	2.530	5.29	2.31	2.98
18	Bharohiya	2.751	0.00	2.62	− 2.62
19	Belghat	2.783	1.63	2.67	− 1.04
20	Gagaha	2.556	2.04	2.35	− 0.31

## Discussion

In this study, the DRI showed a weak and statistically non-significant concordance with block-level dengue incidence in 2025 (Pearson’s *r* = 0.16, *p* = 0.49; Spearman's *ρ* = − 0.003, *p* = 0.99; *R*^2^ = 0.03), and the count-based negative binomial model led to the same conclusion. The bootstrap confidence interval (− 0.41 to 0.53) is wide, and with 20 blocks the study had 80% power only to detect correlations of about 0.59 or larger; a modest true association, therefore, cannot be excluded, but the data provide no evidence that the DRI reproduces the spatial pattern of reported incidence. The most defensible interpretation is that the DRI describes relative spatial susceptibility within the district but has not been validated against incidence. Rather than predicting the number of dengue cases, the DRI was developed as a spatial susceptibility index to identify geographical areas, where environmental, hydrological, and socio-demographic conditions favor dengue transmission [30, 69, 70]. The weak concordance may reflect both the multifactorial nature of dengue transmission and specific limitations of the present data and design, which are examined below.

Several methodological characteristics of the present framework may have limited the strength of spatial concordance. First, the climatic layers carry the largest weights in the ESI (LST and rainfall together 0.70), but rainfall is available only on a 0.25° grid (approximately three by four grid cells across the district) and, therefore, contributes mainly a regional west–east gradient rather than local discrimination between blocks; moreover, 2025 rainfall in the district (mean 689 mm) was well below the long-term normal, so the rainfall pattern of a single deficient year may not represent typical conditions. Perfectly redundant predictors (relative humidity and NDMI) were removed, so that no environmental signal was counted twice (Supplementary Table S14). Second, sensitivity analyses showed that block rankings were stable under equal thematic weights, ± 20% perturbation of thematic weights, ±10% perturbation of individual criterion weights, and use of transmission-season rather than annual rainfall (Spearman's *ρ* ≥ 0.93; Supplementary Tables S16 and S17), suggesting that the subjective AHP weighting is unlikely to explain the weak concordance on its own, although uncertainty in reclassification thresholds was not propagated into the concordance statistics. Third, the choice of denominator strongly affected the apparent concordance: with Census 2011 populations the Pearson correlation appeared significant (*r* = 0.52) only because Chargawan’s census population excludes Gorakhpur city, illustrating how easily a single mis-specified block can drive results when *n* = 20. Fourth, household density still relies on Census 2011, and all climatic and spectral inputs summarize a single year. Finally, uncertainty in the Land Use/Land Cover classification (overall accuracy 78.9%, kappa = 0.74; Supplementary Table S15) propagates into the ESI and, in turn, into the DRI. Collectively, these methodological factors, together with epidemiological complexity, provide a more complete explanation for the weak concordance observed and should guide the interpretation and future refinement of the framework.

Dengue transmission results from dynamic interactions among mosquito vectors, dengue virus, susceptible human populations, environmental conditions, and public health interventions. Accordingly, observed dengue incidence is influenced by factors, including vector abundance, virus serotypes, population immunity, rainfall timing, human mobility, vector-control programs, healthcare-seeking behavior, surveillance quality, reporting completeness, and stochastic outbreak dynamics, all of which vary across space and time. Previous studies likewise demonstrate that environmental variables explain only part of the observed heterogeneity in dengue incidence, because transmission emerges from the interaction of ecological, epidemiological, and social processes rather than climatic conditions alone [30, 51, 69, 71].

Within this context, the ESI, HI, and VI should be interpreted as indicators of dengue transmission potential rather than direct measures of disease occurrence. These indices identify locations, where environmental and socio-demographic conditions favor *Aedes* mosquito survival, breeding, and increased human–vector contact but do not directly capture entomological, virological, behavioral, or surveillance-related processes [72, 73]. The low coefficient of determination observed in this study is, therefore, not unexpected for a susceptibility-based framework, but it also means that the present data do not demonstrate that the DRI captures the determinants of reported incidence. Previous spatial epidemiological studies indicate that integrating environmental suitability with entomological, epidemiological, and socioeconomic information improves dengue risk assessment [3, 30, 51, 71].

The findings should also be interpreted within the context of the ecological study design, in which both framework development and the concordance assessment were based on aggregated block-level dengue incidence and area-level environmental, hydrological, and socio-demographic variables. Consequently, the observed relationships describe population-level spatial patterns rather than individual-level causal associations. Although ecological fallacy cannot be excluded, because substantial heterogeneity may exist within administrative units, ecological analyses remain appropriate for dengue surveillance and public health planning, where vector-control programs, outbreak response, and resource allocation are typically implemented at community and district levels. Accordingly, the proposed framework is intended to identify priority geographical areas for surveillance and intervention rather than estimate individual susceptibility to dengue infection. From a public health perspective, the DRI may help district health authorities structure discussions about, where surveillance, larval source management, and community awareness activities could be strengthened. However, given its weak concordance with reported incidence, it should complement rather than replace surveillance data, and its use for allocating resources would require confirmation with multi-year incidence data and entomological indicators.

Another important consideration is the temporal resolution of the environmental variables used in this study. The DRI was developed from annual environmental conditions to identify areas with persistent spatial suitability for dengue transmission. Although appropriate for mapping long-term spatial susceptibility, this approach does not capture the short-term temporal dynamics that characterize dengue epidemics. Dengue transmission exhibits pronounced seasonality, with fluctuations in rainfall, temperature, and relative humidity influencing mosquito breeding, survival, biting behavior, viral replication, and disease transmission. In addition, increases in dengue incidence often occur several weeks after rainfall because of delays associated with larval development, adult mosquito emergence, the extrinsic incubation period of the virus, and subsequent human transmission. Annual climatic summaries cannot represent these rainfall–dengue lag effects, mosquito generation cycles, seasonal epidemic peaks, or intra-annual climatic variability. This limitation follows directly from two design choices: rainfall was aggregated as the 2025 annual total from IMD daily gridded data, and LST and the spectral indices were derived as 2025 annual mean composites from Landsat-8/9 rather than as a seasonally resolved time series (Table 1; Supplementary Table S18). Replacing annual rainfall with the June–October transmission-season total produced an almost identical block ranking (Spearman's *ρ* = 0.99; Supplementary Table S16), but because monthly block-level case data were not available for this analysis, we did not model lagged or seasonal effects on incidence. We, therefore, restrict the interpretation of the DRI to long-term spatial susceptibility; it should not be used to predict the timing or magnitude of seasonal outbreaks.

The weak concordance between the DRI and observed dengue incidence further highlights that environmental suitability represents only one component of the transmission process. Vector abundance is influenced not only by favorable environmental conditions but also by breeding-site availability, habitat productivity, insecticide resistance, local ecological characteristics, and vector-control interventions. Consequently, areas with similar environmental suitability may exhibit substantially different mosquito densities and transmission potential [74, 75]. Furthermore, established entomological indicators, including the Breteau Index, House Index, and Container Index, provide direct measures of *Aedes* breeding intensity and are widely recognized as indicators of dengue transmission risk. These datasets were unavailable at a spatially continuous district scale and, therefore, could not be incorporated into the present framework. Similarly, viral and host-related factors, including circulating dengue virus serotypes, viral virulence, secondary infections, and population immunity, strongly influence outbreak magnitude and disease severity but cannot be adequately represented using environmental indicators alone [70, 76, 77]. Human behavior, socioeconomic conditions, healthcare accessibility, surveillance quality, reporting completeness, and population mobility further contribute to spatial variation in dengue incidence, emphasizing that observed disease patterns arise from complex interactions among environmental, biological, behavioral, and health-system determinants.

Healthcare accessibility, represented in this framework by Distance to Health Facilities within the Vulnerability Index, warrants particular caution, because it plausibly influences both sides of the DRI–incidence comparison. On one hand, poor healthcare accessibility is hypothesized to increase vulnerability to unmanaged dengue transmission (higher suitability score assigned to greater distance). On the other hand, the observed incidence data used for the concordance assessment are laboratory-confirmed cases reported through the health system; areas that are further from health facilities may under-ascertain and under-report cases regardless of true transmission intensity, while areas close to health facilities (including Gorakhpur city itself) may show disproportionately high observed incidence partly because of better access to diagnosis and reporting rather than genuinely higher transmission. This dual role, whereby a single variable simultaneously represents a hypothesized risk factor and a determinant of case-ascertainment/reporting bias in the outcome, could not be disentangled with the available data and should be considered when interpreting block-level residuals, including the large negative residuals observed for Sardarnagar and Bharohiya, which recorded no laboratory-confirmed cases despite Moderate DRI (Table 8). The concentration of almost half of all reported cases in Chargawan, the block containing Gorakhpur city and its tertiary health facilities, is consistent with this interpretation, although it is also consistent with genuinely higher transmission in the most urbanized block.

## Limitations

Several limitations should be considered when interpreting the findings of this study. First, the concordance assessment was based on laboratory-confirmed dengue surveillance data for a single year (2025) and only 20 administrative blocks. With *n* = 20, the study could reliably detect only correlations of about 0.59 or larger, so the non-significant result does not exclude a weaker true association. Zero counts (Sardarnagar and Bharohiya) may reflect absent reporting rather than absence of transmission, although excluding Bharohiya did not change the results. A single year does not capture interannual variability associated with climatic fluctuations, vector dynamics, viral circulation, or periodic epidemics. The weak and non-significant concordance means that the DRI has not been validated against dengue incidence and should be interpreted as a spatial susceptibility framework rather than as a predictive model. Future studies incorporating multi-year epidemiological data at finer spatial units (e.g., PHC catchments or villages), prospective evaluation, external datasets, and comparisons with statistical or machine-learning approaches would provide a stronger assessment of framework performance and generalizability.

A related limitation concerns the socio-demographic layers. To address the 14-year gap between Census 2011 and the 2025 case data, population density and incidence denominators were based on WorldPop 2025 constrained population estimates, which are modeled by random forest-based dasymetric redistribution of census-based projections [33, 34] and are released as an alpha (R2025A) product that may be revised. Household density was only available from Census 2011 and may misrepresent current residential clustering, particularly in rapidly urbanizing peri-urban blocks. Block-level density values were assigned uniformly within each block, so the VI does not capture within-block variation in population distribution. In addition, Chargawan block contains Gorakhpur city, and case reporting for urban residents may not be fully separable from the rural block; this affects the Chargawan incidence estimate and illustrates the sensitivity of block-level analyses to the choice of denominator.

Third, the environmental component was developed using annual climatic and spectral datasets, with rainfall available only at 0.25° resolution, making the framework suitable for identifying long-term spatial suitability but not short-term epidemic dynamics. The framework does not explicitly capture monthly or weekly climatic variability, seasonal epidemic peaks, mosquito generation cycles, or rainfall–dengue lag effects that influence outbreak timing and intensity. Incorporating higher temporal resolution climatic, epidemiological, and entomological data would facilitate the development of seasonal forecasting and early-warning systems. Fourth, several important determinants of dengue transmission could not be incorporated, because spatially continuous district-level datasets were unavailable. These included entomological indicators (Breteau Index, House Index, and Container Index), vector density, insecticide resistance, circulating dengue virus serotypes, population immunity, household water-storage practices, solid waste management, socioeconomic characteristics, human mobility, healthcare-seeking behavior, and surveillance completeness. The omission of these variables may partly explain the remaining variation between the DRI and observed dengue incidence. Integrating these datasets with GIS and remote-sensing information would strengthen future spatial risk assessment frameworks.

An independent accuracy assessment of the Land Use/Land Cover classification, based on a 30% hold-out sample (*n* = 1,498 pixels), indicated an overall accuracy of 78.9% and a kappa coefficient of 0.74 (substantial agreement; Supplementary Table S15), with class-specific producer's accuracies ranging from 70.7% (Agriculture) to 85.4% (Open Space). This level of classification uncertainty is typical for pixel-based supervised classification of moderate-resolution imagery but nonetheless propagates into the ESI, and in turn into the DRI, given that LULC carries an explicit AHP weight (0.125 within the ESI); future work could evaluate object-based or machine-learning classification approaches to improve LULC accuracy, particularly for the agriculture class.

Finally, the study employed the AHP to derive criterion weights. Although all pairwise comparison matrices satisfied the recommended consistency criterion (CR < 0.10), expert-derived weighting inevitably involves some subjectivity. The use of a multidisciplinary expert panel, independent scoring, consensus review, and consistency testing minimized this limitation; however, alternative weighting schemes may produce different results. Sensitivity analyses showed that block rankings were stable under equal thematic weights and ± 20% thematic-weight perturbation (Spearman's *ρ* = 0.93–1.00; 4–5 of the five highest-ranked blocks retained) and under ±10% perturbation of each criterion weight (*ρ* ≥ 0.99) (Supplementary Tables S16 and S17). However, the equal-interval reclassification thresholds and the Jenks class boundaries are data-dependent, and their uncertainty was not propagated into the concordance statistics. Future research should compare expert-based and data-driven weighting approaches and incorporate Monte Carlo or Bayesian uncertainty analysis to improve the robustness and transparency of spatial dengue risk assessment.

## Conclusions

This study developed a transparent GIS–AHP framework that integrates environmental suitability, hazard, and population vulnerability into a Dengue Risk Index for Gorakhpur district. The DRI showed marked, spatially clustered heterogeneity, with the highest values in Chargawan, which contains Gorakhpur city, and in the central-western blocks. However, its concordance with single-year, block-level dengue incidence was weak and not statistically significant, and the index, therefore, describes relative, long-term spatial susceptibility rather than predicting or explaining reported incidence. The risk classes are relative to this district and are not epidemiologically calibrated. The framework relies on widely available geospatial and demographic data and can be reproduced in other districts, but before it is used to prioritize surveillance or vector-control resources it should be evaluated against multi-year incidence data at finer spatial units and extended with seasonal climatic, entomological, and health-system information.

## Supplementary Information

Supplementary material 1

## Data Availability

The datasets used and/or analysed during the current study are available from the corresponding author on reasonable request.

## Abbreviations

GIS: Geographic Information System
RS: Remote sensing
MCDA: Multi-criteria decision analysis
AHP: Analytic hierarchy process
WLC: Weighted linear combination
DRI: Dengue Risk Index
ESI: Environmental Suitability Index
HI: Hazard Index
VI: Vulnerability Index
DEM: Digital elevation model
CRS: Coordinate reference system
LULC: Land use/land cover
LST: Land surface temperature
RH: Relative humidity
NDVI: Normalized Difference Vegetation Index
NDMI: Normalized Difference Moisture Index
MNDWI: Modified Normalized Difference Water Index
NDBI: Normalized Difference Built-up Index
TWI: Topographic Wetness Index
RMSE: Root mean square error
MAE: Mean absolute error
IRR: Incidence rate ratio
OLS: Ordinary least squares
LM: Lagrange multiplier
WHO: World Health Organization
WHO SEARO: World Health Organization South–East Asia Regional Office
GBD: Global Burden of Disease
IVM: Integrated vector management
PHC: Primary Health Center
CHC: Community Health Center
