# Supplementary material for: An integrated GIS and remote-sensing framework for district-level dengue risk assessment in northern India: development of a spatial Dengue Risk Index and assessment of its spatial concordance with reported incidence
Source: Trop Med Health. 2026 Oct 22;54:225. doi: 10.1186/s41182-026-01096-1 (PMC13688948; doi:10.1186/s41182-026-01096-1)

Supplementary Materials

An integrated GIS and remote sensing framework for district-level dengue risk assessment in northern India: development of a spatial Dengue Risk Index and assessment of its spatial concordance with reported incidence

Supplementary Note S1. Summary of data-processing corrections made during revision

1. Redundant predictors and rainfall source. In the supplied rasters, the ERA5-Land relative humidity layer was an exact linear function of the rainfall layer initially prepared from the same source (pixel-level r = 1.000), so the two layers did not carry independent information. Rainfall was therefore re-derived from the India Meteorological Department (IMD) 0.25° daily gridded rainfall dataset as the 2025 annual total (277–1,033 mm within the district), and relative humidity was excluded. NDBI was the exact negative of NDMI (r = −1.000; both computed from Landsat bands 5 and 6), and NDMI was excluded. ESI weights were re-derived from the 6 × 6 sub-matrix of the original expert matrix (Tables S2–S3).

2. LULC scoring. The LULC raster stores class codes (1 water, 2 built-up, 3 agriculture, 4 vegetation, 5 open space, as defined in the Google Earth Engine classification). These codes had been used directly as suitability scores; they were recoded to the suitability scores stated in Table S1a (water 5, built-up 4, vegetation 3, agriculture 2, open space 1).

3. Direction of distance to river. The distance-to-river raster stores 1.6 km buffer bands (1 = nearest). It is now scored so that the nearest band receives score 5, consistent with the negative relationship stated in Table S11.

4. Aspect. Aspect is now classified into five circular sectors (Table S1a), replacing a linear 0–360° cut in which 1° and 359° fell in opposite classes.

5. Elevation and slope were scored negatively, as stated in Table S11.

6. Grid alignment. Input layers were on three different grids (a geographic 0.00027° grid and two UTM grids offset by non-integer pixels). All layers were resampled to one UTM Zone 44N 30 m reference grid (bilinear for continuous, nearest-neighbour for categorical layers) before overlay.

7. DRI construction and scale. The block DRI values in the previous Table 6 could not be reproduced from the reported ESI, HI, and VI values and weights. The DRI was therefore recomputed pixel-wise as 0.540·ESI + 0.163·HI + 0.297·VI on the continuous 1–5 scale; block values are zonal means (Tables S21, S24).

8. Population denominators. Population density and incidence denominators now use WorldPop 2025 constrained estimates summed to block boundaries (Table S22). The Census 2011 figure for Chargawan excludes Gorakhpur city, and the Census population of Bharohiya was missing in the block attribute table.

9. Case data. Block-level laboratory-confirmed dengue cases were updated to the final 2025 line list (225 cases; Table S20).

10. AHP statistics. All weights and consistency statistics are now computed with the principal-eigenvector method (Table S12).

11. Elevation source. Elevation, slope, aspect, and TWI are derived from the Copernicus Global DEM GLO-30 (30 m), distributed by OpenTopography; earlier text that referred to the SRTM DEM has been corrected.

12. Bharohiya. No dengue cases were recorded for Bharohiya in the 2025 line list; the block was assigned zero cases, and a sensitivity analysis excluding it is reported in Table S23.

Supplementary Table S1. Reclassification criteria, suitability direction, and scientific basis used for standardizing the 14 retained predictor variables and the thematic indices.

Domain	Predictor variable	Native resolution	Scale	Direction	Scientific basis of classification
ESI	Land Surface Temperature (LST)	30 m	1–5	Positive	Temperature regulates Aedes development, survival, biting activity, and dengue virus replication (Brady et al., 2014; Mordecai et al., 2017).
ESI	Rainfall (IMD gridded, 2025 annual total)	0.25° (~25 km)	1–5	Positive	Rainfall creates and replenishes breeding habitats (Lowe et al., 2018; Morin et al., 2013).
ESI	NDVI	30 m	1–5	Positive	Vegetation density influences habitat suitability and local microclimate.
ESI	MNDWI	30 m	1–5	Positive	Surface-water distribution indicates potential breeding habitats.
ESI	NDBI	30 m	1–5	Positive	Built-up environments are associated with Aedes aegypti breeding and human exposure.
ESI	Land Use/Land Cover (LULC)	30 m	1–5	Categorical	Land-cover classes differ in habitat suitability and human–vector contact.
HI	Elevation (DEM)	30 m	1–5	Negative	Low-lying areas accumulate water and drainage.
HI	Slope	30 m	1–5	Negative	Flat terrain retains surface water.
HI	Aspect	30 m	1–5	Circular sectors	South-facing slopes receive more solar radiation (warmer); scored by direction, not degree.
HI	Topographic Wetness Index (TWI)	30 m	1–5	Positive	Indicates potential water accumulation.
HI	Distance to river	Vector (30 m grid)	1–5	Negative	Proximity to rivers increases moisture and breeding habitats.
VI	Population density (WorldPop 2025)	~100 m, aggregated to block	1–5	Positive	Population concentration increases human exposure.
VI	Household density (Census 2011)	Block level	1–5	Positive	Residential clustering enhances human–vector contact.
VI	Distance to health facilities	Vector (30 m grid)	1–5	Positive (farther = more vulnerable)	Poorer access delays diagnosis and case management.
DRI	ESI, HI, VI	30 m	1–5 (continuous)	Positive	Weighted linear combination of thematic indices (AHP weights).

Note: Relative humidity, NDMI, and Percent Vegetation were screened out as redundant (Supplementary Table S14). All thematic indices and the DRI are weighted means of 1–5 scores and therefore remain on the 1–5 scale without further rescaling.

Supplementary Table S1a. Numeric and categorical thresholds used to convert each retained predictor to the 1–5 suitability score (1 = Very Low, 5 = Very High).

Variable	Method, direction	Score 1	Score 2	Score 3	Score 4	Score 5
LST (°C)	Equal interval, positive	22.97 – 27.47	27.47 – 31.97	31.97 – 36.48	36.48 – 40.98	40.98 – 45.48
Rainfall, 2025 annual total (mm)	Equal interval, positive	277 – 428	428 – 580	580 – 731	731 – 882	882 – 1033
NDVI	Equal interval, positive	−0.26 – −0.04	−0.04 – 0.19	0.19 – 0.41	0.41 – 0.64	0.64 – 0.86
MNDWI	Equal interval, positive	−0.56 – −0.31	−0.31 – −0.06	−0.06 – 0.19	0.19 – 0.44	0.44 – 0.69
NDBI	Equal interval, positive	−0.73 – −0.53	−0.53 – −0.33	−0.33 – −0.13	−0.13 – 0.07	0.07 – 0.27

Variable	Method, direction	Score 1	Score 2	Score 3	Score 4	Score 5
Elevation (m)	Equal interval, negative	106.17 – 117.18	95.16 – 106.17	84.14 – 95.16	73.13 – 84.14	62.12 – 73.13
Slope (°)	Equal interval, negative	21.98 – 27.47	16.48 – 21.98	10.99 – 16.48	5.49 – 10.99	0.00 – 5.49
TWI	Equal interval, positive	0.28 – 0.36	0.36 – 0.44	0.44 – 0.53	0.53 – 0.61	0.61 – 0.69
Aspect (°)	Circular sectors	N: 337.5–22.5	NE/NW: 22.5–67.5, 292.5–337.5	E/W: 67.5–112.5, 247.5–292.5	SE/SW: 112.5–157.5, 202.5–247.5	S: 157.5–202.5
LULC	Categorical	Open space	Agriculture	Vegetation	Built-up	Water
Distance to river (km)	Buffer bands, negative	6.4–8.0	4.8–6.4	3.2–4.8	1.6–3.2	0–1.6
Distance to health facility (km)	Buffer bands, positive	0–1.2	1.2–2.4	2.4–3.6	3.6–4.8	4.8–6.0
Population density 2025 (persons/km²)	Jenks (20 blocks), positive	1,058 – 1,346	1,347 – 1,651	1,652 – 2,122	2,123 – 2,468	8,135 (Chargawan)
Household density (households/km²)	Equal interval, positive	99.40 – 125.24	125.24 – 151.08	151.08 – 176.92	176.92 – 202.76	202.76 – 228.60

Note: Equal-interval classes span the observed district minimum to maximum. For negatively scored variables the lowest values receive score 5. Aspect sectors: the north sector is continuous across 0°/360°. Population density classes were derived by Jenks Natural Breaks across the 20 block values; household density by equal intervals. The earlier version of this table labelled all thresholds as Jenks breaks; the thresholds were in fact equal intervals, which is now stated correctly.

Supplementary Table S2. Pairwise comparison matrix for the Environmental Suitability Index (ESI; six retained criteria).

Criteria	LST	Rainfall	NDVI	MNDWI	NDBI	LULC
LST	1	3	8	6	9	4
Rainfall	1/3	1	6	4	7	2
NDVI	1/8	1/6	1	1/3	2	1/4
MNDWI	1/6	1/4	3	1	4	1
NDBI	1/9	1/7	1/2	1/4	1	1/5
LULC	1/4	1/2	4	1	5	1

Note: The matrix is the 6 × 6 sub-matrix of the original expert consensus matrix after removal of relative humidity and NDMI (redundant criteria); expert judgements are unchanged. λmax = 6.224; CI = 0.045; RI = 1.24; CR = 0.036.

Supplementary Table S3. Final AHP weights for the ESI (principal-eigenvector method).

Rank	Predictor variable	AHP weight
1	LST	0.463
2	Rainfall	0.241
3	LULC	0.125
4	MNDWI	0.098
5	NDVI	0.042

Rank	Predictor variable	AHP weight
6	NDBI	0.030

Note: CR = 0.036. For comparison, the original eight-criterion weights were LST 0.341, RH 0.241, rainfall 0.169, LULC 0.087, MNDWI 0.068, NDMI 0.044, NDVI 0.029, NDBI 0.021.

Supplementary Table S4. Pairwise comparison matrix for the Hazard Index (HI).

Criteria	Elevation	Slope	Aspect	TWI	Distance to river
Elevation	1	3	5	1/2	1/3
Slope	1/3	1	3	1/4	1/5
Aspect	1/5	1/3	1	1/6	1/7
TWI	2	4	6	1	2
Distance to river	3	5	7	1/2	1

Note: $\lambda max = 5.225$; CI = 0.056; RI = 1.12; CR = 0.050 (principal-eigenvector method).

Supplementary Table S5. Final AHP weights for the HI.

Rank	Predictor variable	AHP weight
1	TWI	0.373
2	Distance to river	0.331
3	Elevation	0.175
4	Slope	0.080
5	Aspect	0.041

Note: Weights recomputed with the principal-eigenvector method (earlier approximate weights: TWI 0.367, distance to river 0.326, elevation 0.181, slope 0.079, aspect 0.047).

Supplementary Table S6. Pairwise comparison matrix for the Vulnerability Index (VI).

Criteria	Population density	Household density	Distance to health facilities
Population density	1	3	2
Household density	1/3	1	1/2
Distance to health facilities	1/2	2	1

Note: $\lambda max = 3.009$; CI = 0.0046; RI = 0.58; CR = 0.0079.

Supplementary Table S7. Final AHP weights for the VI.

Rank	Predictor variable	AHP weight
1	Population density	0.540

Rank	Predictor variable	AHP weight
2	Distance to health facilities	0.297
3	Household density	0.163

Supplementary Table S8. Pairwise comparison matrix for integrating the thematic indices into the DRI.

Criteria	ESI	HI	VI
ESI	1	3	2
HI	1/3	1	1/2
VI	1/2	2	1

Note: λmax = 3.009; CI = 0.0046; RI = 0.58; CR = 0.0079 (principal-eigenvector method).

Supplementary Table S9. Final AHP weights for constructing the DRI.

Rank	Thematic index	AHP weight
1	Environmental Suitability Index (ESI)	0.540
2	Vulnerability Index (VI)	0.297
3	Hazard Index (HI)	0.163

Note: DRI = 0.540·ESI + 0.163·HI + 0.297·VI (1–5 relative scale).

Supplementary Table S10. Summary of the GIS–AHP–WLC framework used to construct the DRI.

Thematic index	Predictor variables	No.	Weighting	Integration	Output scale
ESI	LST, rainfall, NDVI, MNDWI, NDBI, LULC	6	AHP	WLC	1–5
HI	Elevation, slope, aspect, TWI, distance to river	5	AHP	WLC	1–5
VI	Population density, household density, distance to health facilities	3	AHP	WLC	1–5
DRI	ESI, HI, VI	3	AHP	WLC; Jenks 5 relative classes	1–5

Supplementary Table S11. Characteristics and expected relationship of predictor variables with dengue suitability.

Domain	Predictor	Unit	Data source	Pre-processing	Expected relationship
ESI	LST	°C	Landsat-8/9 TIRS	2025 annual mean composite	Positive
ESI	Rainfall	mm	IMD 0.25° daily gridded rainfall	2025 annual total (sum of daily values); bilinear resampling	Positive
ESI	NDVI	Index	Landsat-8/9	2025 annual mean	Positive
ESI	MNDWI	Index	Landsat-8/9	2025 annual mean	Positive
ESI	NDBI	Index	Landsat-8/9	2025 annual mean	Positive
ESI	LULC	Categorical	Landsat-8/9 (Random Forest)	Post-monsoon composite	Class-specific
HI	Elevation	m	Copernicus DEM GLO-30 (OpenTopography)	—	Negative
HI	Slope	Degrees	Derived from DEM	Terrain analysis	Negative
HI	Aspect	Degrees	Derived from DEM	Circular sectors	Direction-specific (south = highest)
HI	TWI	Index	Derived from DEM	Hydrological modelling	Positive
HI	Distance to river	km	OpenStreetMap	Euclidean distance bands	Negative
VI	Population density	Persons/km²	WorldPop R2025A	Block total / block area	Positive
VI	Household density	Households/km²	Census of India 2011	Block level	Positive
VI	Distance to health facilities	km	OpenStreetMap / Health Department	Euclidean distance bands	Positive (poorer access)

Note: The earlier version listed distance to health facilities as "negative"; the scoring actually applied (and described in the main text) assigns higher vulnerability to greater distance.

Supplementary Table S12. Consistency statistics for the AHP pairwise comparison matrices (principal-eigenvector method).

Matrix	No. of criteria	λmax	CI	RI	CR
Environmental Suitability	6	6.224	0.045	1.24	0.036
Hazard	5	5.225	0.056	1.12	0.050
Vulnerability	3	3.009	0.0046	0.58	0.0079
DRI thematic	3	3.009	0.0046	0.58	0.0079

Note: All CR < 0.10. This table supersedes the previous Tables S12 and S17 (approximate vs eigenvector audit).

Supplementary Table S13. Composition of the multidisciplinary expert panel used for AHP weight derivation.

Discipline	No. of experts	Professional experience (years)
Public Health	2	4–6
Epidemiology	1	5
Climatology	1	6
Environmental Science	1	4
GIS and Remote Sensing	1	3
Medical Geography	1	8
Vector-borne Disease (Entomology)	1	4
Total	8	3–8 (mean 5)

Supplementary Table S14. Predictor redundancy screening across the ESI, HI, and VI domains.

Domain	Pair	Pearson r	Spearman ρ	Decision
ESI	Relative humidity – Rainfall, initial ERA5 layer	1.000	1.000	Removed (RH): identical to the initial ERA5-Land rainfall layer, which was replaced by IMD rainfall
ESI	Relative humidity – Rainfall (IMD)	0.468	0.443	RH not retained (provenance; see Methods)
ESI	NDMI – NDBI	−1.000	−1.000	Removed (NDMI): NDBI = −NDMI algebraically (same bands)
ESI	MNDWI – NDBI	−0.515	−0.364	Retained
ESI	NDVI – NDBI	−0.467	−0.716	Retained
ESI	NDVI – MNDWI	−0.459	−0.140	Retained
ESI	LST – NDBI	0.415	0.359	Retained
ESI	LST – NDVI	−0.209	−0.279	Retained
ESI	LST – Rainfall (IMD)	0.067	0.080	Retained
ESI	Rainfall (IMD) – NDVI	−0.005	−0.005	Retained
ESI	Percent Vegetation – NDVI	0.800	0.950	Removed (PV) in the previous revision
HI	Elevation – Slope (scores)	0.125	0.092	Retained
HI	Elevation – Aspect (scores)	−0.042	−0.044	Retained
HI	Elevation – TWI (scores)	0.226	0.212	Retained

Domain	Pair	Pearson r	Spearman ρ	Decision
HI	Elevation – Distance to river (scores)	0.118	0.143	Retained
HI	Slope – Aspect (scores)	0.005	0.003	Retained
HI	Slope – TWI (scores)	0.213	0.229	Retained
HI	Slope – Distance to river (scores)	0.012	0.007	Retained
HI	Aspect – TWI (scores)	−0.059	−0.040	Retained
HI	Aspect – Distance to river (scores)	0.016	0.011	Retained
HI	TWI – Distance to river (scores)	−0.004	0.005	Retained
VI	Population density – Household density (block level, n = 20)	0.535	0.334	Retained
VI	Population density – Distance to health facilities (block level, n = 20)	0.169	−0.444	Retained
VI	Household density – Distance to health facilities (block level, n = 20)	0.024	0.039	Retained

Note: ESI and HI correlations are pixel-level (random sample of 200,000 district pixels); ESI values use raw variables, HI values use 1–5 scores. Exact collinearity (|r| = 1.000) led to removal of one variable of each pair.

Supplementary Table S14a. Association of LULC class with continuous ESI predictors (eta-squared, correlation ratio).

Continuous variable	Eta-squared	Eta
MNDWI	0.454	0.674
NDVI	0.283	0.532
NDBI	0.125	0.353
LST	0.041	0.202
Rainfall	0.003	0.052

Note: LULC class codes follow the Google Earth Engine classification: 1 = water, 2 = built-up, 3 = agriculture, 4 = vegetation, 5 = open space; suitability scores were then assigned as in Table S1a.

Supplementary Table S15. Confusion matrix and accuracy assessment of the Land Use/Land Cover classification (independent 30% hold-out sample, n = 1,498 pixels; rows = reference, columns = classified).

Reference \ Classified	Water	Built-up	Agriculture	Vegetation	Open space	Row total	Producer's accuracy
Water	224	2	15	17	28	286	78.32%

Reference \ Classified	Water	Built-up	Agriculture	Vegetation	Open space	Row total	Producer’s accuracy
Built-up	0	246	60	2	0	308	79.87%
Agriculture	6	61	210	18	2	297	70.71%
Vegetation	12	7	15	238	26	298	79.87%
Open space	27	1	2	15	264	309	85.44%
Column total	269	317	302	290	320	1,498	–
User’s accuracy	83.27%	77.60%	69.54%	82.07%	82.50%	–	–

Note: Overall accuracy = 78.91% (1,182 of 1,498 pixels correctly classified); kappa coefficient = 0.736 (substantial agreement). Matrix exported directly from the Random Forest classification output (Google Earth Engine errorMatrix); the previous version of this table contained transcription errors in the cell counts, but the reported accuracy statistics were correct and are unchanged.

Supplementary Table S16. Sensitivity of block rankings and spatial concordance to the thematic (ESI/HI/VI) weights.

Scenario	W(ESI)	W(HI)	W(VI)	Spearman ρ vs baseline ranking	Top-5 blocks retained	Pearson r with incidence	Spearman ρ with incidence
Baseline AHP	0.540	0.163	0.297	1.000	5/5	0.164	-0.003
Equal weights	0.333	0.333	0.333	0.926	5/5	0.187	0.070
ESI +20%	0.585	0.147	0.268	0.973	4/5	0.156	-0.014
ESI −20%	0.484	0.183	0.333	0.973	5/5	0.169	0.044
VI +20%	0.510	0.154	0.336	0.976	5/5	0.166	0.049
VI −20%	0.574	0.173	0.253	0.965	4/5	0.156	-0.003
HI +20%	0.523	0.189	0.288	0.998	4/5	0.166	0.016
HI −20%	0.558	0.135	0.307	1.000	5/5	0.161	-0.003
8-criterion ESI (RH, NDMI retained)	0.540	0.163	0.297	0.770	3/5	0.241	0.145
Rainfall as Jun–Oct total (transmission season)	0.540	0.163	0.297	0.992	5/5	0.164	-0.001

Note: ±20% scenarios change one thematic weight by 20% and renormalize the three weights to sum to one. The 8-criterion scenario retains relative humidity and NDMI with the original eight-criterion AHP weights. The transmission-season scenario replaces annual rainfall with the June–October 2025 IMD total (220–904 mm). Incidence uses WorldPop 2025 denominators (n = 20).

Supplementary Table S17. Criterion-level sensitivity: effect of ±10% perturbation of each criterion weight (renormalized within its index) on block-level DRI.

Index	Criterion	Weight	Mean DRI change −10% (%)	ρ vs baseline (−10%)	Top-5 retained (−10%)	Mean DRI change +10% (%)	ρ vs baseline (+10%)	Top-5 retained (+10%)
ESI	LST	0.463	0.085	0.998	4/5	−0.073	0.998	5/5
ESI	Rainfall	0.241	−0.220	0.997	5/5	0.214	0.997	4/5
ESI	NDVI	0.042	−0.070	0.998	4/5	0.073	1.000	5/5
ESI	MNDWI	0.098	0.308	0.998	4/5	−0.297	1.000	5/5

Index	Criterion	Weight	Mean DRI change −10% (%)	ρ vs baseline (−10%)	Top-5 retained (−10%)	Mean DRI change +10% (%)	ρ vs baseline (+10%)	Top-5 retained (+10%)
ESI	NDBI	0.030	−0.054	0.998	4/5	0.058	1.000	5/5
ESI	LULC	0.125	−0.035	0.998	4/5	0.039	1.000	5/5
HI	Elevation	0.175	−0.074	1.000	5/5	0.076	1.000	5/5
HI	Slope	0.080	−0.072	1.000	5/5	0.075	1.000	5/5
HI	Aspect	0.041	0.012	1.000	5/5	−0.008	1.000	5/5
HI	TWI	0.373	0.217	1.000	5/5	−0.197	1.000	5/5
HI	Distance to river	0.331	−0.068	1.000	5/5	0.068	0.998	4/5
VI	Population density	0.540	0.069	0.997	4/5	−0.057	0.994	5/5
VI	Household density	0.163	−0.112	0.997	4/5	0.113	1.000	5/5
VI	Distance to health facilities	0.297	0.053	0.995	5/5	−0.046	0.998	4/5

Note: Supersedes the previous Table S23, whose weights did not correspond to the reported AHP weights.

Supplementary Table S18. Spatial data sources, resolution, and acquisition details.

Variable	Type	Data source	Spatial / temporal resolution	Period
LST	Environmental	Landsat-8/9 TIRS (Collection 2 Level-2)	30 m / annual mean composite	2025
Rainfall	Climatic	India Meteorological Department (IMD) high-resolution daily gridded rainfall (Pai et al., 2014)	0.25° × 0.25° (~25 km) / daily, summed to annual total (mm)	2025
NDVI	Environmental	Landsat-8/9 OLI (bands 4, 5)	30 m / annual mean	2025
MNDWI	Environmental	Landsat-8/9 OLI (bands 3, 6)	30 m / annual mean	2025
NDBI	Environmental	Landsat-8/9 OLI (bands 5, 6)	30 m / annual mean	2025
LULC	Environmental	Landsat-8/9; Random Forest; GLC-FCS30D reference	30 m / post-monsoon composite	2025
Elevation, slope, aspect, TWI	Topographic	Copernicus Global DEM GLO-30, distributed by OpenTopography (doi:10.5069/G9028PQB)	30 m / static	2021 release
Distance to river	Hydrological	OpenStreetMap	Vector → 30 m grid	2025 extraction

Variable	Type	Data source	Spatial / temporal resolution	Period
Distance to health facilities	Accessibility	OpenStreetMap / District Health Department	Vector → 30 m grid	2025 extraction
Population	Socio-demographic	WorldPop Global2 constrained estimates of total number of people per grid square, R2025A version v1 (Bondarenko et al., 2025; doi:10.5258/SOTON/WP00839). Random Forest-based dasymetric redistribution; WGS84; people per pixel; alpha release	3 arc-seconds (~100 m at the equator), summed to block	2025
Household density	Socio-demographic	Census of India	Block level	2011
Dengue cases	Epidemiological	District Health Surveillance System (IDSP), Gorakhpur	Block level, laboratory-confirmed	Jan–Dec 2025
Relative humidity, NDMI (screened out)	—	ERA5-Land; Landsat-8/9	—	2025
Rainfall, initial layer (replaced)	—	ERA5-Land (identical to relative humidity layer, r = 1.000)	—	2025

Supplementary Table S19. Summary statistics of predictor variables within Gorakhpur district (30 m pixels unless stated).

Variable	Unit	Min	Max	Mean	SD
LST	°C	22.96	45.60	32.69	2.30
Rainfall (2025 annual total, IMD)	mm	277.1	1033.4	688.9	203.0
NDVI	—	−0.26	0.86	0.43	0.11
MNDWI	—	−0.56	0.69	−0.36	0.11
NDBI	—	−0.78	0.29	−0.09	0.09
Elevation	m	61.44	121.63	76.89	4.69
Slope	°	0.00	29.46	1.35	1.60
Aspect	°	0.00	360.00	—	—
TWI	—	0.28	0.69	0.44	0.09
Population density 2025 (blocks)	persons/km²	1,058	8,135	1,889	1,520
Household density (blocks)	households/km²	99.48	228.60	163.20	32.58
Relative humidity (screened out)	%	46.78	51.90	48.30	1.42
NDMI (screened out)	—	−0.29	0.78	0.09	0.09

Note: Aspect is circular; its arithmetic mean is not meaningful and is not reported.

Supplementary Table S20. Block-level dengue cases, 2025 population, observed and regression-estimated incidence (per 100,000).

S. No	Block	Mean DRI	Cases 2025	Population 2025 (WorldPop)	Observed incidence	Estimated incidence	Residual
1	Campierganj	2.757	8	244,720	3.27	2.63	0.64
2	Jungle Kaudia	2.992	13	181,219	7.17	2.97	4.20
3	Chargawan	3.351	110	1,935,207	5.68	3.48	2.20
4	Pali	2.820	2	156,151	1.28	2.72	−1.44
5	Pipraich	2.815	11	300,961	3.65	2.72	0.94
6	Sahjanwa	3.085	3	313,729	0.96	3.10	−2.15
7	Piprauli	2.987	4	291,605	1.37	2.96	−1.59
8	Khorabar	2.778	9	388,454	2.32	2.66	−0.35
9	Sardarnagar	2.877	0	295,218	0.00	2.80	−2.80
10	Khajni	2.876	9	257,505	3.50	2.80	0.69
11	Bansgaon	2.687	17	247,608	6.87	2.53	4.33
12	Brahmpur	2.174	3	235,169	1.28	1.80	−0.53
13	Kauriram	2.516	2	229,561	0.87	2.29	−1.42
14	Uruwa	2.837	1	333,837	0.30	2.75	−2.45
15	Gola	2.721	4	235,818	1.70	2.58	−0.89
16	Barhalganj	2.450	10	264,041	3.79	2.20	1.59
17	Bhathat	2.530	12	226,833	5.29	2.31	2.98
18	Bharohiya	2.751	0	175,725	0.00	2.62	−2.62
19	Belghat	2.783	3	184,168	1.63	2.67	−1.04
20	Gagaha	2.556	4	196,336	2.04	2.35	−0.31
	Total		225	6,693,865	3.36		

Note: OLS: incidence = −1.301 + 1.427 × mean DRI (n = 20). Case counts from the 2025 block-wise line list (225 cases); the single case reported under "Gorakhpur Urban" was assigned to Chargawan, which contains the city; Derwa, Karauta and Ragar Ganj reported no cases; Bharohiya had no cases recorded in the line list and is recorded as 0 (sensitivity analysis excluding Bharohiya in Table S23).

Supplementary Table S21. Zonal statistics of the DRI raster by block (30 m pixels).

Block	Pixels	Min	Max	Mean	Median	SD
Campierganj	250,633	1.875	3.606	2.757	2.771	0.224
Jungle Kaudia	149,430	2.276	3.613	2.992	2.992	0.161
Chargawan	264,305	2.531	4.280	3.351	3.346	0.252
Pali	154,689	1.951	3.557	2.820	2.821	0.178
Pipraich	156,523	2.224	3.482	2.815	2.816	0.177

Block	Pixels	Min	Max	Mean	Median	SD
Sahjanwa	179,373	2.233	3.974	3.085	3.093	0.183
Piprauli	179,117	2.128	3.895	2.987	2.972	0.256
Khorabar	174,890	2.006	3.655	2.778	2.774	0.195
Sardarnagar	140,726	2.264	3.522	2.877	2.870	0.160
Khajni	186,272	1.939	3.537	2.876	2.885	0.222
Bansgaon	183,102	1.986	3.455	2.687	2.678	0.180
Brahmpur	202,841	1.425	3.119	2.174	2.162	0.201
Kauriram	175,702	1.686	3.526	2.516	2.511	0.186
Uruwa	265,739	2.041	3.611	2.837	2.846	0.189
Gola	157,260	2.089	3.586	2.721	2.714	0.190
Barhalganj	226,975	1.699	3.353	2.450	2.441	0.188
Bhathat	172,769	1.945	3.250	2.530	2.506	0.205
Bharohiya	132,320	2.109	3.464	2.751	2.761	0.184
Belghat	176,463	1.934	3.796	2.783	2.769	0.201
Gagaha	155,780	1.963	3.276	2.556	2.552	0.156

Supplementary Table S22. Comparison of Census 2011 and WorldPop 2025 denominators and resulting incidence (per 100,000).

Block	Cases 2025	Population, Census 2011 (shapefile)	Population, WorldPop 2025	Incidence (Census)	Incidence (WorldPop)	Area (km²)	Population density 2025
Campierganj	8	274,914	244,720	2.91	3.27	231.3	1,058
Jungle Kaudia	13	235,235	181,219	5.53	7.17	134.5	1,347
Chargawan	110	208,230	1,935,207	52.83	5.68	237.9	8,135
Pali	2	144,080	156,151	1.39	1.28	140.9	1,108
Pipraich	11	188,645	300,961	5.83	3.65	141.8	2,123
Sahjanwa	3	151,488	313,729	1.98	0.96	163.1	1,923
Piprauli	4	190,262	291,605	2.10	1.37	161.2	1,809
Khorabar	9	203,081	388,454	4.43	2.32	157.4	2,468
Sardarnagar	0	178,005	295,218	0.00	0.00	128.0	2,307
Khajni	9	196,763	257,505	4.57	3.50	168.4	1,529
Bansgaon	17	176,523	247,608	9.63	6.87	164.8	1,503
Brahmpur	3	193,681	235,169	1.55	1.28	184.3	1,276
Kauriram	2	176,345	229,561	1.13	0.87	158.5	1,448

Block	Cases 2025	Population, Census 2011 (shapefile)	Population, WorldPop 2025	Incidence (Census)	Incidence (WorldPop)	Area (km²)	Population density 2025
Uruwa	1	202,021	333,837	0.49	0.30	239.7	1,393
Gola	4	167,583	235,818	2.39	1.70	142.7	1,652
Barhalganj	10	182,977	264,041	5.47	3.79	208.2	1,268
Bhathat	12	193,535	226,833	6.20	5.29	157.2	1,443
Bharohiya	0	missing	175,725	—	0.00	121.0	1,452
Belghat	3	195,776	184,168	1.53	1.63	161.1	1,143
Gagaha	4	185,525	196,336	2.16	2.04	141.2	1,390

Note: The Census figure for Chargawan (208,230) refers to the rural block only, whereas the block polygon (237.9 km²) contains Gorakhpur city (WorldPop 1,935,207). The Census population for Bharohiya was missing (0) in the shapefile attribute table, whose area and density fields had been copied from Jungle Kaudia.

Supplementary Table S23. Spatial concordance statistics under the primary analysis and sensitivity analyses.

Analysis set	n	Pearson r	p	Bootstrap 95% CI	Spearman ρ	p	Kendall τ	R²	RMSE	MAE	Shapiro–Wilk residuals W (p)	Breusch–Pagan χ² (p)	Max Cook's D	NB IRR per 0.1 DRI (95% CI)
Primary: WorldPop 2025 denominators, all blocks	20	0.164	0.491	−0.41 to 0.53	−0.003	0.990	−0.026	0.027	2.107	1.758	0.929 (0.149)	0.76 (0.382)	Chargawan (0.37)	1.05 (0.91–1.21); p = 0.498
WorldPop, excluding Chargawan	19	−0.019	0.940	−0.52 to 0.38	−0.142	0.562	−0.117	0.000	2.071	1.696	0.891 (0.034)	0.52 (0.470)	Jungle Kaudia (0.40)	0.98 (0.81–1.20); p = 0.878
WorldPop, excluding Bharohiya (no reported cases)	19	0.166	0.497	−0.41 to 0.55	−0.030	0.904	−0.053	0.028	2.072	1.735	0.934 (0.208)	0.86 (0.353)	Chargawan (0.35)	1.05 (0.92–1.20); p = 0.485
Census 2011 denominators, excluding Bharohiya	19	0.524	0.021	−0.39 to 0.80	−0.005	0.983	−0.006	0.274	9.637	6.720	0.800 (0.001)	5.92 (0.015)	Chargawan (4.04)	1.25 (1.09–1.42); p = 0.001
Census 2011, excluding Bharohiya and Chargawan	18	−0.065	0.799	−0.51 to 0.32	−0.183	0.468	−0.124	0.004	2.424	2.065	0.911 (0.091)	0.15 (0.702)	Brahmpur (0.47)	0.98 (0.82–1.16); p = 0.804

Note: Poisson dispersion ratio (primary analysis) = 4.59. The apparently strong Census-based correlation is driven entirely by Chargawan (Cook's D = 6.08), whose Census denominator excludes Gorakhpur city.

Supplementary Table S24. Zonal statistics of the ESI, HI, and VI rasters by block (1–5 scale).

Block	ESI mean	HI mean	VI mean	ESI range	HI range	VI range
Campierganj	3.017	3.335	1.963	1.911–4.061	1.371–4.825	1.000–2.678
Jungle Kaudia	3.154	3.702	2.306	2.034–4.129	2.308–5.000	1.540–3.381
Chargawan	2.921	3.075	4.284	1.683–4.338	1.296–5.000	3.158–5.000
Pali	3.236	3.468	1.709	2.140–4.129	1.828–5.000	1.000–2.515
Pipraich	2.342	3.277	3.418	1.637–3.405	1.591–4.825	3.109–4.000
Sahjanwa	3.186	3.404	2.725	2.140–4.647	1.552–5.000	2.243–3.594
Piprauli	2.845	3.625	2.894	1.416–4.159	2.098–5.000	2.243–3.460
Khorabar	2.208	3.432	3.452	1.175–3.604	1.572–5.000	2.782–4.297
Sardarnagar	2.322	3.313	3.643	1.187–3.405	1.842–4.959	2.782–4.460
Khajni	3.050	3.502	2.216	1.881–4.129	1.591–5.000	1.703–2.891
Bansgaon	2.697	3.486	2.229	1.880–3.887	1.882–5.000	1.866–3.054
Brahmpur	2.060	3.661	1.564	1.175–3.405	2.213–5.000	1.163–2.515
Kauriram	2.426	3.372	2.208	1.175–3.898	1.767–5.000	1.703–3.054
Uruwa	2.970	3.423	2.273	1.795–4.139	1.882–5.000	1.703–3.054
Gola	2.667	3.229	2.539	1.848–3.761	1.552–5.000	2.243–3.430
Barhalganj	2.597	3.454	1.632	1.564–3.856	1.767–5.000	1.163–2.351
Bhathat	2.377	3.358	2.351	1.511–3.341	1.452–4.825	1.540–3.218
Bharohiya	3.059	3.261	1.911	2.089–3.948	1.591–4.838	1.540–2.787
Belghat	3.118	3.455	1.803	2.121–4.381	1.591–4.838	1.327–2.515
Gagaha	2.553	3.494	2.045	1.638–3.645	1.767–5.000	1.703–2.891

Supplementary Table S25. Pearson (lower triangle) and Spearman (upper triangle) correlation matrix of candidate ESI predictors (pixel level).

Variable	LST	Rainfall (IMD)	Rainfall, initial ERA5 layer	Relative humidity	NDVI	NDMI	MNDWI	NDBI
LST	1	0.080	−0.287	−0.287	−0.279	−0.359	−0.017	0.359
Rainfall (IMD)	0.067	1	0.443	0.443	−0.005	−0.052	0.012	0.052
Rainfall, initial ERA5 layer	−0.225	0.468	1	1.000	0.177	0.086	−0.051	−0.086
Relative humidity	−0.225	0.468	1.000	1	0.177	0.086	−0.051	−0.086

Variable	LST	Rainfall (IMD)	Rainfall, initial ERA5 layer	Relative humidity	NDVI	NDMI	MNDWI	NDBI
NDVI	−0.209	−0.005	0.182	0.182	1	0.716	−0.140	−0.716
NDMI	−0.415	−0.042	0.111	0.111	0.467	1	0.364	−1.000
MNDWI	−0.198	0.002	−0.021	−0.021	−0.459	0.515	1	−0.364
NDBI	0.415	0.042	−0.111	−0.111	−0.467	−1.000	−0.515	1

Note: Random sample of 200,000 district pixels. Relative humidity, NDMI, and the initial ERA5-Land rainfall layer are shown to document their exclusion or replacement.

Supplementary Table S26. Pearson (lower) and Spearman (upper) correlations among vulnerability predictors (block level, n = 20).

Variable	Population density	Household density	Distance to health facilities
Population density	1	0.334	−0.444
Household density	0.535	1	0.039
Distance to health facilities	0.169	0.024	1

Note: Distance to health facilities is summarized as the block mean of the 1–5 distance-band score.

Supplementary Table S27. Pearson (lower) and Spearman (upper) correlations among hazard scores (pixel level).

Variable	Elevation	Slope	Aspect	TWI	Distance to river
Elevation	1	0.092	−0.044	0.212	0.143
Slope	0.125	1	0.003	0.229	0.007
Aspect	−0.042	0.005	1	−0.040	0.011
TWI	0.226	0.213	−0.059	1	0.005
Distance to river	0.118	0.012	0.016	−0.004	1

Note: Raw-variable Pearson correlations: elevation–slope 0.29, elevation–TWI −0.30, slope–TWI −0.73.

Supplementary Table S28. Global Moran’s I and Lagrange multiplier (LM) tests for spatial dependence.

Variable	Weights	Moran’s I	E[I]	Permutation p (9,999)	LM-error (p)	LM-lag (p)
Block mean DRI (n = 20)	queen	0.217	−0.053	0.048		
Incidence, WorldPop (n = 20)	queen	−0.125	−0.053	0.626		
Incidence, Census (n = 19)	queen	0.009	−0.056	0.260		
Block mean ESI	queen	0.602	−0.053	0.000		
Block mean VI	queen	0.258	−0.053	0.029		

Variable	Weights	Moran's I	E[I]	Permutation p (9,999)	LM-error (p)	LM-lag (p)
Block mean HI	queen	0.027	−0.053	0.586		
OLS residuals, primary (n = 20)	queen	−0.166		0.444	1.03 (0.310)	0.81 (0.368)
OLS residuals, excl. Chargawan	queen	−0.267		0.166	2.28 (0.131)	2.31 (0.129)
OLS residuals, Census (n = 19)	queen	0.059		0.359	0.12 (0.732)	0.01 (0.911)
Block mean DRI (n = 20)	knn4	0.319	−0.053	0.003		
Incidence, WorldPop (n = 20)	knn4	−0.040	−0.053	0.925		
Incidence, Census (n = 19)	knn4	−0.034	−0.056	0.671		
Block mean ESI	knn4	0.639	−0.053	0.000		
Block mean VI	knn4	0.260	−0.053	0.014		
Block mean HI	knn4	−0.073	−0.053	0.889		
OLS residuals, primary (n = 20)	knn4	−0.084		0.799	0.33 (0.567)	0.18 (0.670)
OLS residuals, excl. Chargawan	knn4	−0.157		0.414	1.08 (0.299)	1.11 (0.293)
OLS residuals, Census (n = 19)	knn4	−0.042		0.901	0.08 (0.779)	0.38 (0.540)

Note: queen = queen contiguity with 60 m snapping tolerance, row-standardized; knn4 = four nearest block centroids, row-standardized. Because residual spatial dependence was not significant, spatial lag or error models were not required.

Supplementary Table S29. Dengue cases and pooled incidence by DRI risk class of the block mean (2025).

DRI class	Blocks	Block names	Cases	Population 2025	Pooled incidence /100,000	Share of cases
Very Low	1	Brahmpur	3	235,169	1.28	1.3%
Low	4	Kauriram, Barhalganj, Bhathat, Gagaha	28	916,772	3.05	12.4%
Moderate	11	Campierganj, Pali, Pipraich, Khorabar, Sardarnagar, Khajni, Bansgaon, Uruwa, Gola, Bharohiya, Belghat	64	2,820,165	2.27	28.4%
High	3	Jungle Kaudia, Sahjanwa, Piprauli	20	786,553	2.54	8.9%
Very High	1	Chargawan	110	1,935,207	5.68	48.9%

Note: Kruskal–Wallis test of block incidence across classes: $H = 2.75$, $p = 0.600$ (descriptive; very small groups).

Supplementary Figure S1. Relationship between block mean DRI and observed dengue incidence in 2025 (per 100,000, WorldPop 2025 denominators; n = 20 blocks), with fitted least-squares line.

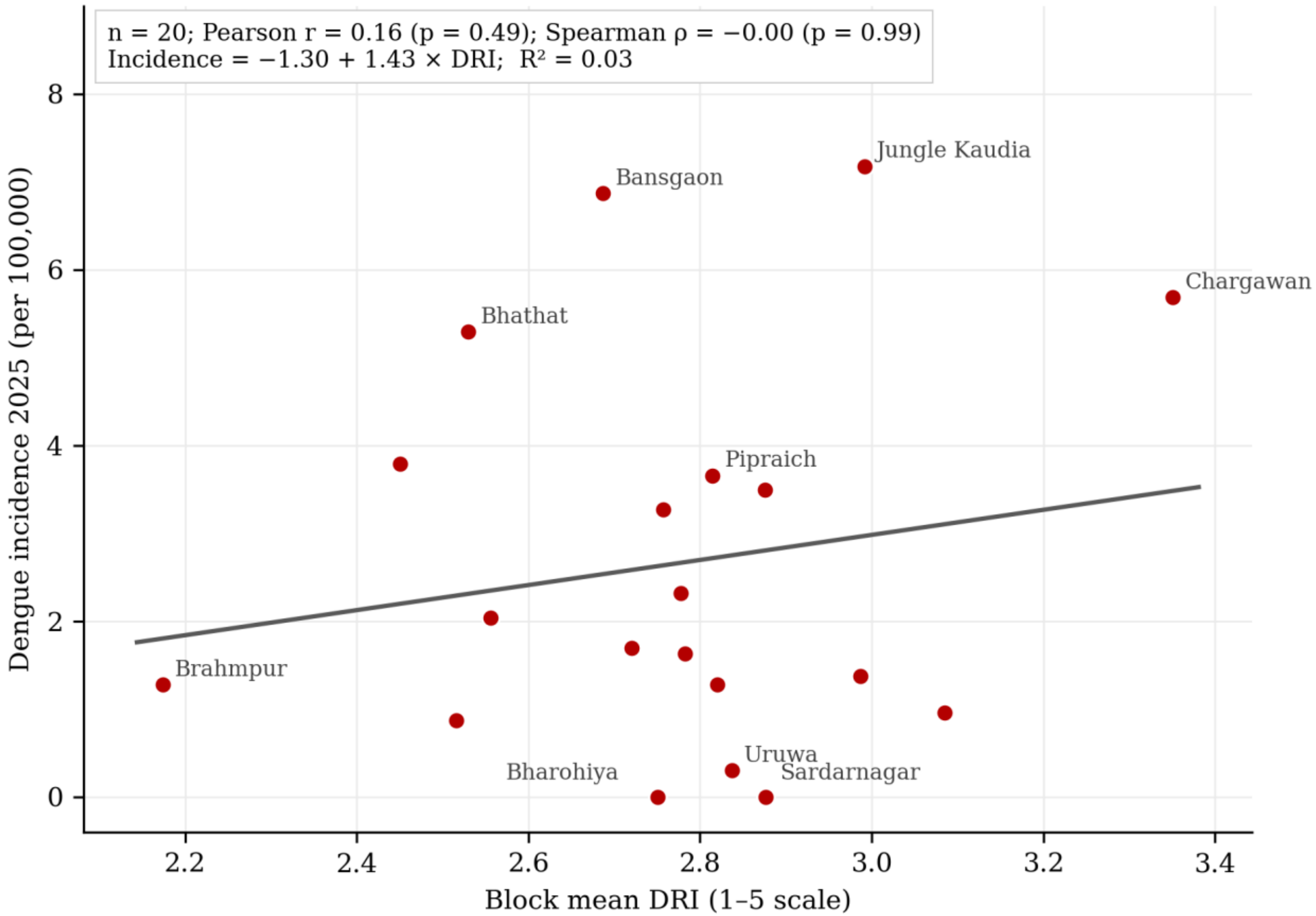

Supplement: Supplementary file 1 [file 41182_2026_1096_MOESM1_ESM.pdf]
